# Programmed initiation and enhancement of cGAS/STING pathway for tumour immunotherapy via tailor‐designed ZnFe_2_O_4_‐based nanosystem

**DOI:** 10.1002/EXP.20230061

**Published:** 2023-11-10

**Authors:** Jing Yang, Yuping He, Meng Zhang, Chenglin Liang, Tongtong Li, Tianjiao Ji, Mali Zu, Xu Ma, Zhenzhong Zhang, Chun Liang, Qixu Zhang, Youbai Chen, Lin Hou

**Affiliations:** ^1^ Key Laboratory of Targeting Therapy and Diagnosis for Critical Diseases, School of Pharmaceutical Sciences Zhengzhou University Zhengzhou China; ^2^ CAS Key Laboratory for Biomedical Effects of Nanomaterials and Nanosafety National Center for Nanoscience and Technology Beijing China; ^3^ Center of Materials Science and Optoelectronics Engineering University of Chinese Academy of Sciences Beijing China; ^4^ Department of Plastic and Reconstructive Surgery Chinese PLA General Hospital Beijing China; ^5^ Department of Plastic Surgery University of Texas MD Anderson Cancer Center Texas USA

**Keywords:** cGAS‐STING pathway, glutathione responsive, tumour immunotherapy, ZnFe_2_O_4_‐based nanosystem

## Abstract

The cyclic guanosine monophosphate‐adenosine monophosphate synthase (cGAS)/stimulator of interferon genes (STING) signalling pathway has been a promising target for anticancer immunity, but rationally activating and enhancing this pathway in tumour cells is critical. Herein, a glutathione sensitive ZnFe_2_O_4_‐based nanosystem is developed to programmatically initiate and enhance the STING signalling pathway in tumour cells. The prepared ZnFe_2_O_4_ nanoparticles were coated with cancer cell membrane (CCM), which enabled the nanosystem target tumour cells. In tumour cells, ZnFe_2_O_4_ nanoparticles could be disintegrated by responding to high level glutathione, and the released Fe^3+^ generated reactive oxygen species to induce the DNA leakage into the cytoplasm to stimulate cGAS. Then Zn^2+^ promoted cGAS‐DNA phase separation to intensify the cGAS enzymatic activity. In addition, the low dose encapsulation of paclitaxel (PTX) acting as an antimitotic agent (ZnFe_2_O_4_‐PTX@CCM) ensured the sustained activation of cGAS/STING pathway. The in vitro and in vivo results confirmed that ZnFe_2_O_4_‐PTX@CCM elevated the cGAS/STING activity, promoted dendritic cell maturation, increased cytotoxic T lymphocyte and natural killer cells infiltration, eventually inhibiting the tumour progress and postoperative recurrence. This study provided feasible references on constructing STING activation nanosystem for tumour immunotherapy.

## Graphical abstract

Tailor‐designed ZnFe_2_O_4_‐based nanosystem can respond to the tumour microenvironment and stimulate anti‐tumour immune responses by enhancing the cGAS/STING pathway. Moreover, it remodels immunosuppressive microenvironment, and effectively inhibits tumour growth as well as postoperative recurrence.

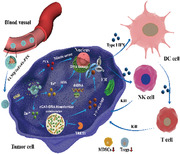

## 1 INTRODUCTION

Activation of the cyclic guanosine monophosphate‐adenosine monophosphate synthase (cGAS)/stimulator of interferon genes (STING) pathway has been a promising strategy of cancer immunotherapy. In brief, after cGAS senses the cytosolic DNA in tumour cells, STING is activated to mediate type I interferon production and proinflammatory cytokine secretion, thus inducing innate immune responses and bridging to adaptive immunity.^[^ ^1^, ^2^ ^]^ However, double stranded DNA (dsDNA) is normally absent in the cytoplasm,^[^ ^3^ ^]^ which limits the cGAS/STING pathway activation.^[^ ^4^ ^]^ Therefore, triggering the exposure of DNA to the cytosolic DNA sensor (cGAS) play a pivotal role in priming cGAS/STING signalling pathway.

Types of strategies, such as generating intracellular reactive oxygen species (ROS), can induce DNA damage and lead to the DNA leakage into the cytoplasm,^[^ ^5^, ^6^, ^7^, ^8^ ^]^ which possesses the potential to activate cGAS/STING axis.^[^ ^9^, ^10^, ^11^, ^12^ ^]^ However, when DNA is leaked into the cytoplasm, it is generally degraded by TREX1 (an exonuclease), resulting in the non‐binding to cGAS.^[^ ^13^, ^14^, ^15^ ^]^ Zhou et al. discovered that cGAS phase separation can resist TREX1‐mediated DNA degradation by forming liquidlike droplets to trap TERX1.^[^ ^16^ ^]^ Therefore, the effective initiation of cGAS should be based on the presence of cytosolic DNA and maintenance of cGAS enzymatic activity that can be intensified by cGAS‐DNA phase separation.

Recently, it is found that with the progression of cell mitosis, cytoplasmic cGAS can be recruited to the nucleus and subsequently nucleosomes bind with cGAS, thus resulting in the decrease of cGAS enzymatic activity and promoting the immune escape.^[^ ^17^, ^18^, ^19^ ^]^ Christian et al. reported that prolonged mitotic arrest could facilitate cGAS‐mediated cell death.^[^ ^20^ ^]^ Inspired by these vital findings, we hypothesize that integrating the induction of cytosolic DNA, improvement of cGAS‐DNA phase separation, and introduction of antimitotic agent possesses a great potential to enhance cGAS/STING pathway.

**FIGURE 1:**
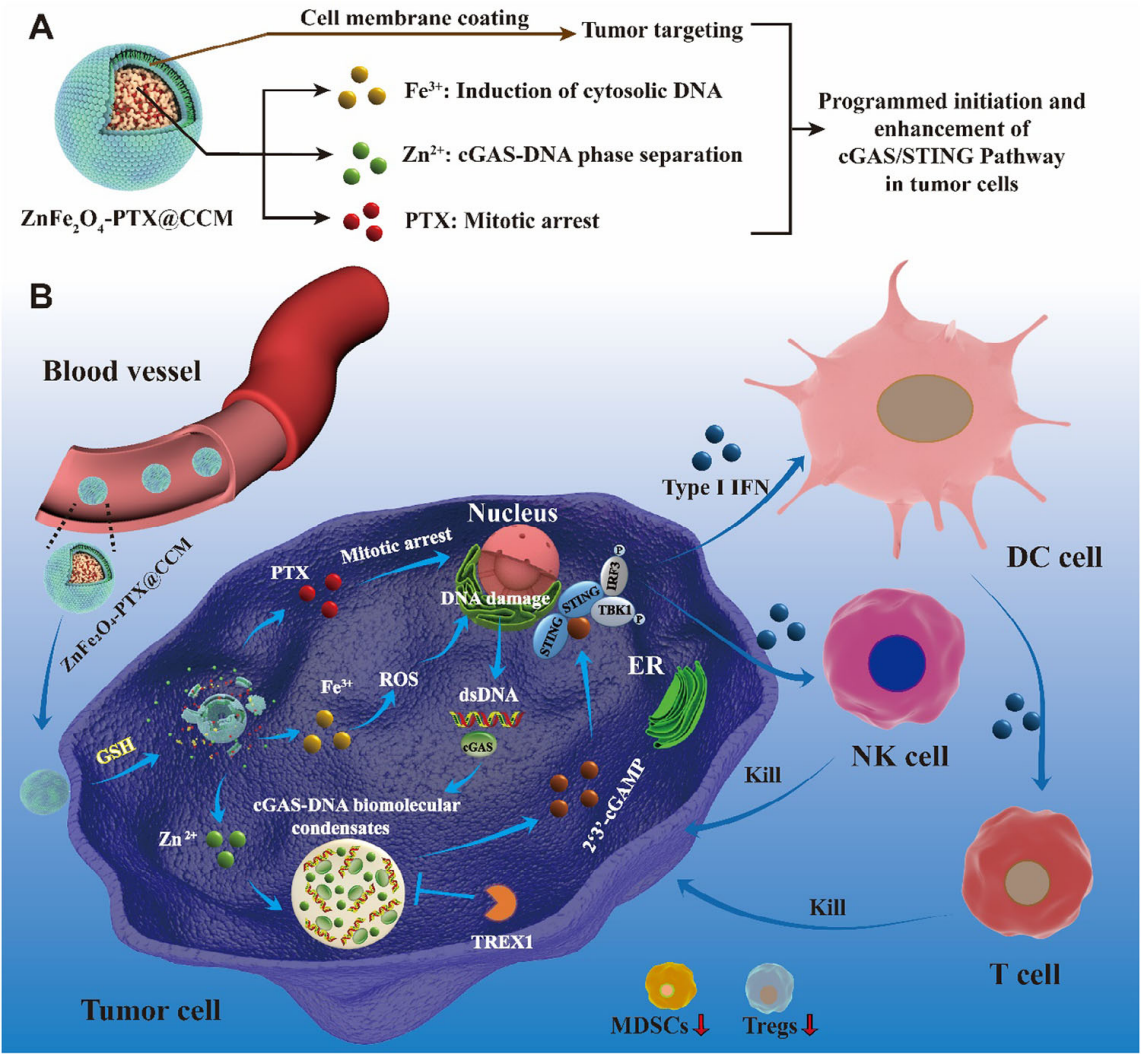
Schematic illustration for antitumor activity of ZnFe_2_O_4_‐PTX@CCM by enhancing cGAS/STING pathway. (A) Components of ZnFe_2_O_4_‐PTX@CCM and their functions. (B) The working mechanisms of ZnFe_2_O_4_‐PTX@CCM in cGAS/STING pathway activation and tumour immunotherapy.

In recent years, tumour immunotherapy mediated by nanomaterials has attracted much attention.^[^ ^21^, ^22^ ^]^ Herein, we constructed tumour intracellular microenvironment responsive ZnFe_2_O_4_ nanoparticles loading low‐dose paclitaxel (PTX) (ZnFe_2_O_4_‐PTX) to programmatically initiate cGAS/STING pathway (Figure 1A). Importantly, the ZnFe_2_O_4_ nanoparticles exhibited glutathione (GSH) sensitivity and possessed mesoporous structure to encapsulate PTX. In brief, the ZnFe_2_O_4_ nanoparticle would release Fe^3+^ and Zn^2+^ in the tumour cytoplasm by responding to the high level of GSH, during which process, Fe^3+^ induced intracellular ROS to achieve DNA leakage and Zn^2+^ promoted cGAS‐DNA phase separation as well as enhanced the cGAS enzyme activity.^[^ ^23^ ^]^ In addition, the released PTX inhibited the tumour cell mitosis, which further strengthened the activation of cGAS/STING pathway (Figure 1A). To achieve the tumour targeting, the ZnFe_2_O_4_‐PTX nanoparticles were coated with cancer cell membrane (CCM) to form the final nanosystem ZnFe_2_O_4_‐PTX@CCM. Therefore, the enhanced activation of cGAS/STING pathway was expected to promote dendritic cell (DC) maturation, natural killer cell (NK) infiltration, cytotoxic T lymphocyte (CTL) activation, and reduce immunosuppressive cells in tumour microenvironment (TME) (Figure 1B).

## 2 RESULTS AND DISCUSSION

### 2.1 Preparation and characterization of ZnFe_2_O_4_‐PTX@CCM

**FIGURE 2:**
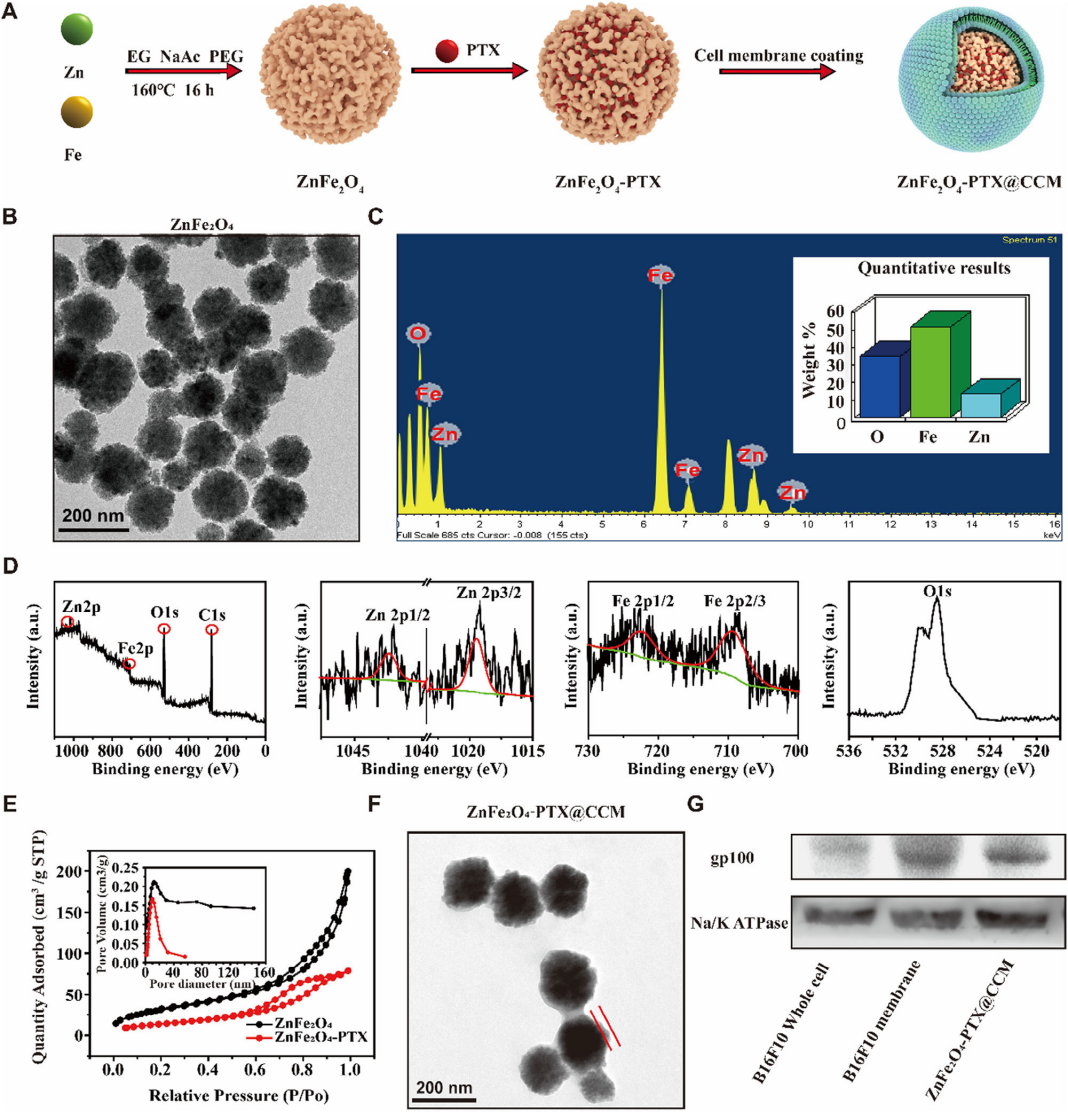
Preparation and characterization of ZnFe_2_O_4_‐PTX@CCM. (A) Schematic illustration of ZnFe_2_O_4_‐PTX@CCM preparation. (B) TEM image of ZnFe_2_O_4_. (C) EDS spectrum of ZnFe_2_O_4_. (D) XPS spectra of the ZnFe_2_O_4_ and XPS peak resolution analysis of Zn 2p, Fe 2p, and O 1s. (E) N_2_ absorption–desorption isotherms and pore‐size distribution curve of ZnFe_2_O_4_ and ZnFe_2_O_4_‐PTX. (F) TEM image of ZnFe_2_O_4_‐PTX@CCM. The area within the red line represents the cell membrane coating. (G) gp100 expression on B16F10 cells, B16F10 cell membrane, and ZnFe_2_O_4_‐PTX@CCM by WB analysis.

ZnFe_2_O_4_ was synthesized using a one‐step solvothermal method (Figure 2A). Uniform and spherical ZnFe_2_O_4_ with a size about 156.70 nm and mesoporous structure was observed through transmission electron microscopy (TEM) (Figure 2B). Energy dispersive spectroscopy (EDS) spectra demonstrated a strong correlation of Zn, Fe, and O elements (Figure 2C) in ZnFe_2_O_4_ nanoparticles. X‐ray photoelectron spectroscopy (XPS) was also tested. As shown in Figure 2D, peaks at binding energies of 1042.40 and 1019.20 eV were assigned to Zn 2p1/2 and Zn 2p3/2, indicating that Zn principally existed in the state of Zn^2+^. In addition, the Fe 2p peaks at 722.60 and 709.50 eV were associated with Fe^3+^. These data confirmed the structure and component of ZnFe_2_O_4_.

In order to verify whether ZnFe_2_O_4_ possesses the capacity to load drugs, the N_2_ adsorption/desorption isotherms were detected. From the results in Figure 2E and Table S1, we determined that ZnFe_2_O_4_ had a surface area of 115.68 m^2^ g^−1^ and an average pore size of 10.69 nm, ensuring the space for drug encapsulation. In comparison, the corresponding parameters decreased significantly after PTX incorporation (the surface area of ≈52.89 nm, average pore size of ≈8.02 nm). According to Figure S1, the absorption peak of PTX at 227 nm was tested in ZnFe_2_O_4_‐PTX, further revealing the successful drug loading. Moreover, the drug loading content of PTX in ZnFe_2_O_4_‐PTX was 50.58%, which was calculated using ultraviolet‐visible spectrophotometry.

After constructing ZnFe_2_O_4_‐PTX nanoparticles, the nanosystem camouflaged with cancer cell membrane (ZnFe_2_O_4_‐PTX@CCM) was prepared via extrusion approach. TEM images in Figure 2F exhibited that ZnFe_2_O_4_‐PTX@CCM possessed a thin corona with the thickness of about 8.00 nm. Figure S2 showed that the particle size increased from 158.70 ± 8.70 nm (ZnFe_2_O_4_‐PTX) to 180.90 ± 15.60 nm (Figure S2A), and the zeta potential reduced from −2.60 ± 0.90 mV (ZnFe_2_O_4_‐PTX) to −31.67 ± 0.70 mV (Figure S2B). Furthermore, the representative protein (gp100) was observed in ZnFe_2_O_4_‐PTX@CCM by western blotting (WB) analysis (Figure 2G). All phenomenon as above suggested the successful membrane decoration. In addition, the particle size and zeta potential of ZnFe_2_O_4_‐PTX@CCM (Figure S3) remained almost unchanged in phosphate‐buffered saline (PBS) (pH 7.4) within 24 h, indicating of the good stability of this formulation.

### 2.2 In vitro evaluation on the responsiveness of ZnFe_2_O_4_‐PTX@CCM

**FIGURE 3:**
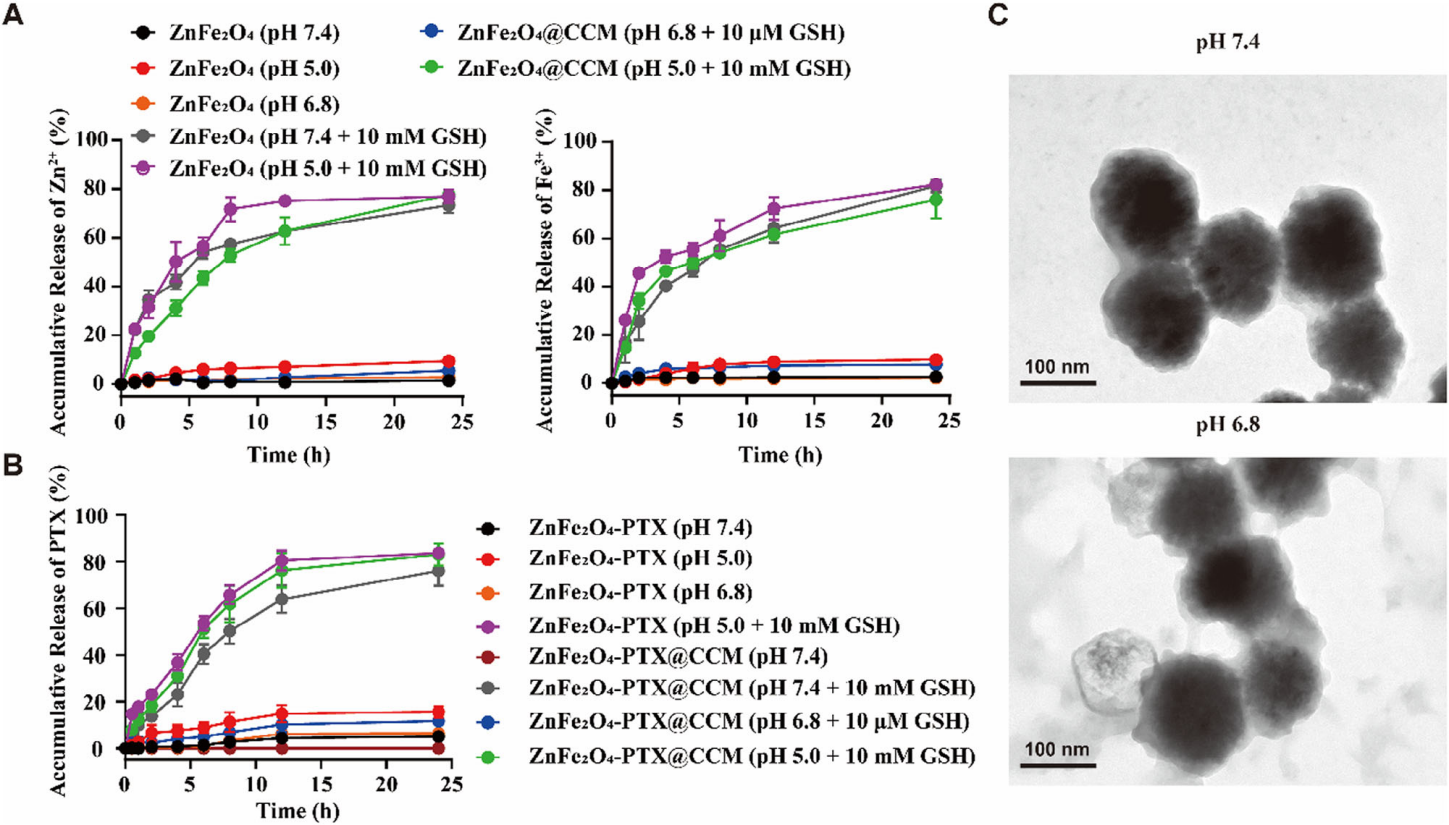
In vitro evaluation on the responsiveness of ZnFe_2_O_4_‐PTX@CCM. (A) Release profiles of Zn^2+^ and Fe^3+^ in PBS buffers (different pH) with or without GSH (*n =* 3, mean ± SD). (B) PTX release profile in different PBS buffer with or without GSH (*n =* 3, mean ± SD). (C) TEM images of ZnFe_2_O_4_‐PTX@CCM in solutions with different pH for 12 h. ZnFe_2_O_4_‐PTX@CCM could maintain the integrity at pH 7.4 and pH 6.8.

Some bimetallic oxide nanosystems having Fe_2_O_4_^2−^ (eg. MnFe_2_O_4_@MOF) were reported to possess glutathione peroxidase‐like activity,^[^ ^24^, ^25^ ^]^ which exhibit GSH‐responsiveness in tumour cells (because of much higher level GSH in tumour cells than that in normal cells as well as extracellular matrix).^[^ ^26^, ^27^ ^]^ Therefore, we investigated the GSH‐triggered release of Zn^2+^ and Fe^3+^ behaviours. As shown in Figure 3A, the accumulation release of Zn^2+^ and Fe^3+^ from ZnFe_2_O_4_@CCM in PBS buffer (pH 5.0) containing 10 mm GSH reached ≈77.69% and ≈75.99% at 24 h, respectively. Similarly, the release of Zn^2+^ and Fe^3+^ from ZnFe_2_O_4_ reached ≈76.99% and ≈82.36%. In comparison, the release content in other conditions without or with low GSH level was less than ≈15%, which indicated the GSH responsiveness and the ion‐producing effect of ZnFe_2_O_4_@CCM. In addition, the PTX release profile from ZnFe_2_O_4_‐PTX@CCM and ZnFe_2_O_4_‐PTX was studied.

The release behaviour of PTX was consistent with that of Zn^2+^ and Fe^3+^ (Figure 3B). In detail, ZnFe_2_O_4_‐PTX exhibited greatly delayed release behaviour with less than 20% of PTX liberated in PBS buffer (pH 7.4, 6.8 or 5.0), and ZnFe_2_O_4_‐PTX@CCM presented the lowest PTX release amount, indicating that ZnFe_2_O_4_ was stable under conditions without or with low GSH level and CCM could further protect drugs from leakage. In comparison, ≈80% of PTX was released from ZnFe_2_O_4_‐PTX@CCM and ZnFe_2_O_4_‐PTX in PBS buffer containing 10 mm GSH at 12 h, confirming the GSH‐triggered drug release. Notably, CCM decoration protected ZnFe_2_O_4_‐PTX from premature leakage before entering tumour cells, probably because the structure of ZnFe_2_O_4_‐PTX@CCM maintained the integrity at pH 7.4 and pH 6.8 (shown as TEM images in Figure 3C).

### 2.3 Evaluations on the cell uptake and lysosome escape of ZnFe_2_O_4_‐PTX@CCM

**FIGURE 4:**
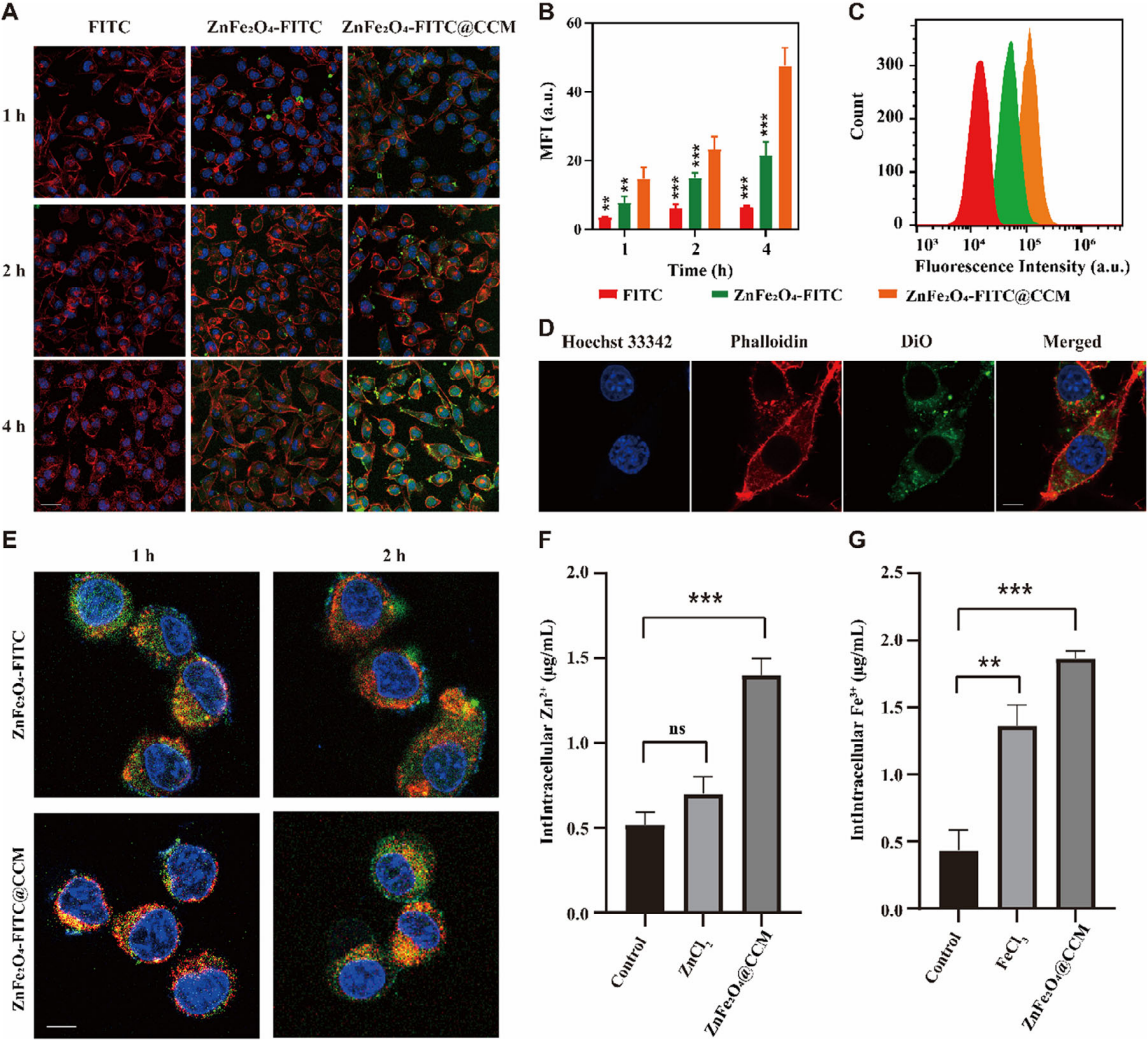
Cell uptake and lysosome escape of ZnFe_2_O_4_‐PTX@CCM. (A) Cellular uptake of FITC, ZnFe_2_O_4_‐FITC and ZnFe_2_O_4_‐FITC@CCM in B16F10 cells via CLSM image. Cell nuclei was stained with Hoechst 33342 (blue fluorescence). Cytoskeleton was stained with YF@555 Dye Phalloidin (red fluorescence), scale bar = 25 μm. (B) The average cellular green fluorescence intensity of each group in (A) (*n =* 3, mean ± SD). (C) Cellular internalization via flow cytometry. (D) CLSM images of DiO‐labelled ZnFe_2_O_4_‐PTX@CCM internalized by B16F10 cells. Cell nuclei was stained with Hoechst 33342 (blue). Cytoskeleton was stained with YF@555 Dye Phalloidin (red fluorescence), scale bar = 8 μm. (E) Lysosome escape of ZnFe_2_O_4_‐FITC@CCM in B16F10 cell. Representative image of Lyso‐tracker red staining the lysosome, and Hoechst 33342 (blue) staining the nuclei, scale bar = 8 μm. (F,G) Intracellular accumulation of Zn (F) and Fe (G) ions (*n =* 3, mean ± SD). *p < 0.05, **p < 0.01, ***p < 0.001, by analysis of ANOVA with Turkey's post‐hoc test.

Cellular uptake was assessed on B16F10 cells, and samples were labelled with fluorescein isothiocyanate (FITC). Compared with the ZnFe_2_O_4_‐FITC group, cells treated with ZnFe_2_O_4_‐FITC@CCM showed stronger green fluorescence signal, which indicated that the CCM decoration increased the internalization of ZnFe_2_O_4_‐FITC (Figure 4A,B). Quantitative analysis by flow cytometry was consistent with the CLSM results (Figure 4C). To investigate the mechanism of the cell uptake, the CCM layer of ZnFe_2_O_4_@CCM were labelled with 3,3′‐Dioctadecyloxacarbocyanine perchlorate (DiO). The DiO signal emerged in the cell rather than the cytoskeleton (Phalloidin labelling) of B16F10, which suggested that the ZnFe_2_O_4_@CCM entered tumour cells through endocytosis rather than membrane fusion (Figure 4D). Moreover, the ZnFe_2_O_4_‐FITC@CCM entered lysosomes (yellow fluorescent signals in Figure 4E, the co‐localization of FITC‐labelled nanoparticles and lysotracker red) in the first hour. However, the green fluorescence intensity in the cytoplasm of the treatment group at 2 h was higher than that at 1 h groups (Figure 4E), indicating ZnFe_2_O_4_‐based nanoparticles could escape from lysosomes. The lysosome escape property is probably attributed to the slightly released Zn^2+^, which can influence the integrity of lysosomes.^[^ ^28^ ^]^

Once entering the cytoplasm, ZnFe_2_O_4_‐PTX@CCM was expected to thoroughly disintegrate under high level of intracellular GSH. Accordingly, the intracellular ion accumulation was detected by inductive coupled plasma emission spectrometer (ICP). As shown in Figure 4F,G, compared with the untreated group, the intracellular Zn^2+^ and Fe^3+^ content for ZnFe_2_O_4_‐PTX@CCM increased 2.73 and 4.85 times, respectively, which were much higher than free Zn^2+^ and Fe^3+^ groups. We also observed the continuous increase of Zn^2+^ in the cytoplasm after ZnFe_2_O_4_‐PTX@CCM treatment, further confirming the disintegration of ZnFe_2_O_4_‐PTX@CCM (Movie S1).

### 2.4 ZnFe_2_O_4_‐PTX@CCM can enhance cGAS/STING pathway

**FIGURE 5:**
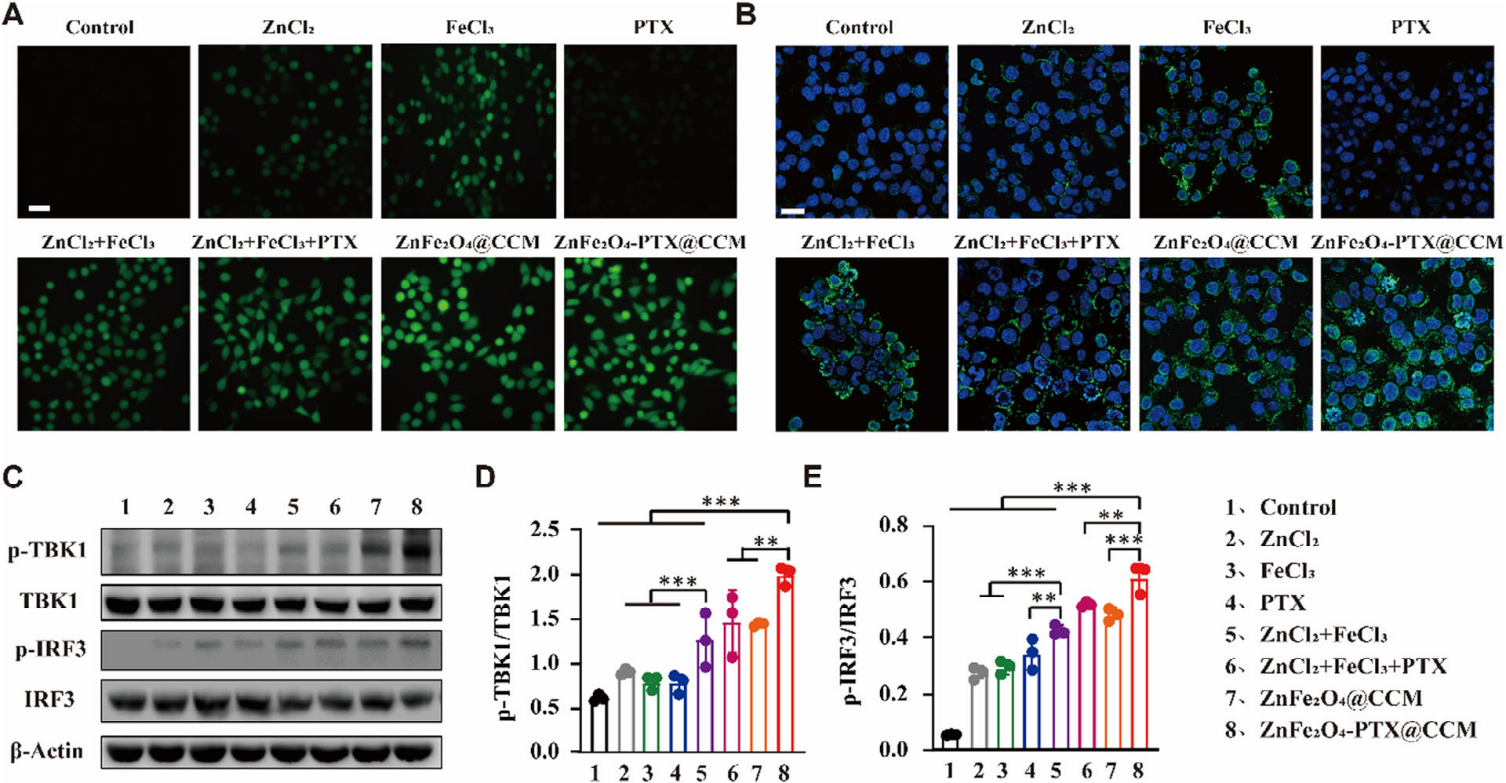
ZnFe_2_O_4_‐PTX@CCM can enhance cGAS/STING signal. (A) After B16F10 cells were treated with different preparations, intracellular ROS was detected by fluorescence microscope. The green fluorescence is generated by oxidized ROS probe, scale bar = 100 μm. (B) CLSM images of the leakage of double‐stranded DNA in the cytoplasm of B16F10 cells treated with different preparations. Green: cytoplasmic dsDNA, blue: cell nucleus, scale bar = 25 μm. (C) WB analysis of related proteins expression levels for cGAS/STING pathway in B16F10 tumour cells treated with different preparations. (D,E) Semiquantitative analysis of the WB bands of (C). Data are presented as mean ± SD (*n* = 3). *p < 0.05, **p < 0.01, ***p < 0.001, by analysis of ANOVA with Turkey's post‐hoc test.

Since the released Fe^3+^ could produce ROS in tumour cells through Fenton reaction and induce DNA damage, intracellular ROS level was detected using fluorescein 2,7‐dichlorodiacetate (DCFH‐DA) probe. It was observed that ZnFe_2_O_4_@CCM and ZnFe_2_O_4_‐PTX@CCM groups demonstrated the strongest fluorescent intensity, followed by ZnCl_2_+FeCl_3_, ZnCl_2_+FeCl_3_+PTX, FeCl_3_, ZnCl_2_, and PTX groups, suggesting that CCM encapsulation increased the intracellular Fe^3+^ amount and facilitated ROS generation (Figure 5A, Figure S4). It has been proved that Zn^2+^ can promote cGAS‐DNA phase separation and increase the enzymatic activity of cGAS.^[^ ^23^, ^29^ ^]^ Therefore, we examined the expression of cGAS‐STING pathway‐associated proteins in B16F10 cells that incubated with different concentrations of ZnCl_2_. As shown in Figure S5, the cGAS‐STING signalling pathway presents a Zn^2+^ concentration‐dependent enhancement. Furthermore, Zn^2+^(20 μm)synergistically elevated the Fe^3+^‐mediated ROS level, probably owing to the lysosomes and mitochondria damage.^[^ ^28^, ^30^ ^]^ Subsequently, the presence of cytoplasmic dsDNA was determined by confocal laser scanning microscopy (CLSM), and consistent results with that of ROS test were obtained in Figure 5B and Figure S6. Compared with all other groups, ZnFe_2_O_4_@CCM and ZnFe_2_O_4_‐PTX@CCM groups exhibited the most dsDNA in the cytoplasm, laying the foundation for cGAS/STING signalling activation.

In order to confirm the activation of cGAS/STING pathway, the related downstream proteins, such as TANK‐binding kinase 1 (TBK1), phosphorylates TBK1 (p‐TBK1), interferon regulatory factor 3 (IRF3) and phosphorylates IRF3 (p‐IRF3) were detected by western blot (WB) (Figure 5C–E). It was found that the expression of p‐TBK1 and p‐IRF3 in B16F10 cells treated with FeCl_3_ alone increased slightly, probably because the DNA in the cytoplasm was easily degraded by TREX1.^[^ ^31^ ^]^ The augmented cGAS/STING pathway signals in the ZnCl_2_+FeCl_3_ group was attributed to the enhanced ROS level and the phase separation of cGAS‐dsDNA induced by Zn^2+^, which restricted TREX1 and inhibited dsDNA degradation.^[^ ^16^, ^23^, ^28^ ^]^ ZnFe_2_O_4_@CCM group exhibited stronger expression of these markers owing to the CCM decoration. In addition, the antimitotic agent PTX can avoid cytoplasmic cGAS into the nucleus and increase the binding of cGAS with dsDNA,^[^ ^20^ ^]^ leading to the enhancement of cGAS/STING pathway after combination with Zn^2+^ and Fe^3+^. Notably, ZnFe_2_O_4_‐PTX@CCM dramatically upregulated the expression of p‐TBK1 and p‐IRF3.

It was observed that ZnFe_2_O_4_‐PTX@CCM did not show obvious toxicity on tumour cells and normal cells (HUVEC cells) (Figure S7). These results demonstrated that the antitumor potency of ZnFe_2_O_4_‐PTX@CCM were not resulted from the directly cell killing, but only took effect on tumour cells owing to the high level GSH responsiveness.

### 2.5 In vivo tumour targeting capability and therapeutic efficacy of ZnFe_2_O_4_‐PTX@CCM

**FIGURE 6:**
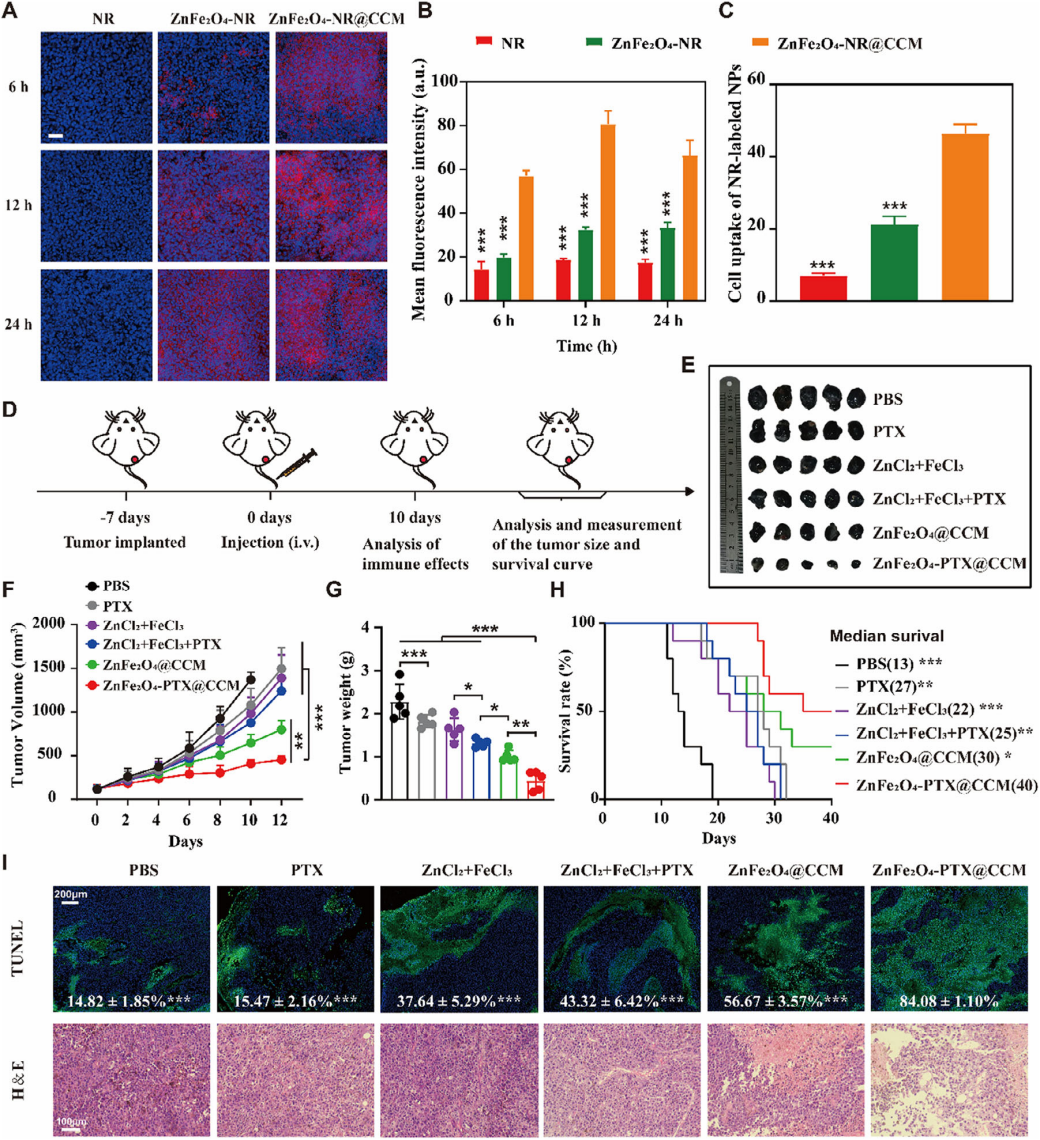
In vivo antitumor effect of ZnFe_2_O_4_‐PTX@CCM. (A) Biodistribution of samples in tumour tissue. Nuclei are shown in blue and NRs are shown in red. Tumour tissue was obtained at 6, 12, and 24 h after injection, scale bar = 50 μm. (B) Quantitative calculation of fluorescent intensity of NR from (A) (*n =* 3, mean ± SD). (C) Analysis of cellular uptake of NR‐labelled nanoparticles by tumour cells in vivo by flow cytometry (*n =* 3, mean ± SD). (D) The regimen of animal experiment. (E) Photograph of the excised tumours at the endpoint. (F) Tumour growth trends of B16F10 tumour‐bearing mice after various treatments (*n =* 10, mean ± SD). (G) Changes in tumour weight of mice after the determined treatments (*n =* 10, mean ± SD). (H) The survival curves of mice that received different treatments (*n =* 10), overall survival curves were generated using the Kaplan–Meier method and estimated by the long rank‐test. (I) TUNEL and H&E staining of tumour sections after different treatments. *p < 0.05, **p < 0.01, ***p < 0.001, by analysis of ANOVA with Turkey's post‐hoc test, compared to ZnFe_2_O_4_‐PTX@CCM.

Tumour targeting capability of formulations is crucial to achieve desired antitumor effect, and in vivo distribution of ZnFe_2_O_4_‐PTX@CCM was evaluated via immunofluorescence staining. Formulations were labelled with Nile red (NR) for visualization. As shown in Figure 6A,B and Figure S8, compared with the control groups, ZnFe_2_O_4_‐NR@CCM demonstrated the specific tumour site location with low distribution in other tissues. The fluorescence intensity of ZnFe_2_O_4_‐NR@CCM reached the highest at 12 h after injection, and maintained visible even at 24 h, indicating that ZnFe_2_O_4_‐NR@CCM possessed tumour targeting and retention effect (Figure 6A,B).

Furthermore, we detected the uptake of ZnFe_2_O_4_‐NR@CCM in mouse tumour cells by flow cytometry. It was found that 46.40% of ZnFe_2_O_4_‐NR@CCM was taken up by tumour cells (Figure 6C), much higher than in the other groups. This phenomenon was owing to the homologous targeting effect of CCM coating, and signified that ZnFe_2_O_4_@CCM could facilitate the endocytosis into tumour cells, potentially leading to effective antitumor therapy.

Subsequently, the antitumor effect of ZnFe_2_O_4_‐PTX@CCM was evaluated on B16F10 tumour‐bearing mice according to the regimen (Figure 6D). Mice were randomly divided into 6 groups and treated by different formulations including PBS, PTX, ZnCl_2_+FeCl_3_, ZnCl_2_+FeCl_3_+PTX, ZnFe_2_O_4_@CCM and ZnFe_2_O_4_‐PTX@CCM, respectively. As shown in Figure 6E–G, ZnFe_2_O_4_‐PTX@CCM exhibited the strongest tumour inhibition ability. Compared with the PBS group, the tumour inhibition rate of ZnFe_2_O_4_‐PTX@CCM was as high as 80.42%, which was significantly better than that of the ZnFe_2_O_4_@CCM treatment group. By contrast, tumour volume of PTX and ZnCl_2_+FeCl_3_ groups grew in a similar manner as PBS group. Although ZnCl_2_+FeCl_3_+PTX group retarded the tumour growth to some extent, the tumour volume still increased quickly with time extended. This result indicated that PTX played an important part in the overall antitumor synergism, but the fast clearance and non‐targeting property of free Zn^2+^, Fe^3+^ or PTX limited their effects. ZnFe_2_O_4_‐PTX@CCM that integrated above elements in the targeting system could conduce to the optimized therapy.

Moreover, the tumoricidal effect of ZnFe_2_O_4_‐PTX@CCM was identified by terminal deoxynucleotidyl transferase dUTP nick end labelling (TUNEL) and hematoxylin and eosin (H&E) staining. The TUNEL images in Figure 6I showed that the ZnFe_2_O_4_‐PTX@CCM caused a large area of apoptosis with the highest cellular apoptosis of ≈84.08%. Maximum dead cells, bulk necrosis and acellular regions were also observed in H&E results of ZnFe_2_O_4_‐PTX@CCM treatment, proving the effective antitumor activity.

In addition, the ZnFe_2_O_4_‐PTX@CCM treatment significantly prolonged the survival period with the median survival time (40 days), compared with PBS (13 days), PTX (27 days), ZnCl_2_+FeCl_3_ (22 days), ZnCl_2_+FeCl_3_+PTX (25 days), and ZnFe_2_O_4_@CCM (30 days) (Figure 6H), further confirming the enhanced efficacy of ZnFe_2_O_4_‐PTX@CCM on B16F10 tumour‐bearing mice. Moreover, tumour development can cause the spleen enlargement, and the enlarged spleen can become hyperfunctioning and fragile in texture, leading to dysfunction of spleen. After treatment, ZnFe_2_O_4_@CCM group did not emerge this phenomenon compared with other groups (Figure S9), which further proved the efficiency of the designed formulation.

The biological safety was determined through extensive toxicological analysis. There were no significant changes in the body weight among various treatment groups (Figure S10). H&E staining images showed no obvious morphological damage or inflammatory injury in major organs (i.e. heart, liver, spleen, lung, and kidney) after treatment with ZnFe_2_O_4_‐PTX@CCM (Figure S11A). Blood biochemical parameters of ZnFe_2_O_4_‐PTX@CCM group, such as the levels of important liver and kidney function markers, remained within the normal ranges, indicating of negligible systemic toxicity (Figure S11B). Taken together, ZnFe_2_O_4_‐PTX@CCM possessed good biocompatibility and biosafety.

### 2.6 ZnFe_2_O_4_‐PTX@CCM enhanced immune cell infiltration and pro‐inflammatory cytokine secretion via improving cGAS/STING pathway

**FIGURE 7:**
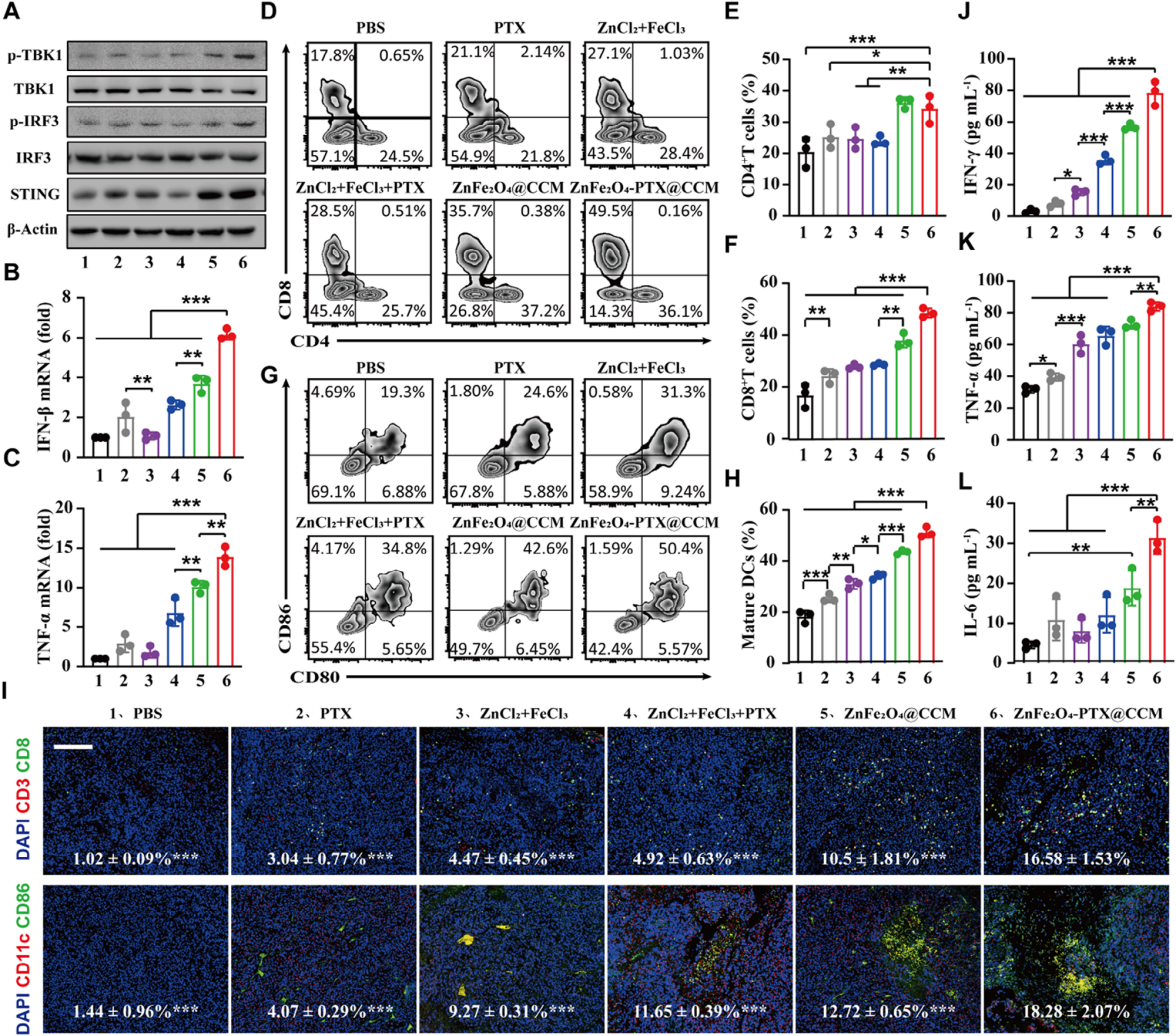
ZnFe_2_O_4_‐PTX@CCM enhanced the infiltration of immune cells and the secretion of pro‐inflammatory cytokines by enhancing the cGAS/STING pathway. (A) Expression analysis of cGAS/STING signalling pathway‐related protein expression in tumour tissues after treatment with different preparations. (B,C) mRNA expression levels of IFN‐β and TNF‐α in tumour tissues (*n =* 3, mean ± SD). (D–F) Flow cytometry examination of the intratumor infiltration of CD4^+^ and CD8^+^ T cells (gated on CD3^+^ T cells) (*n =* 3, mean ± SD). (G,H) The maturation of DCs in tumours measured by flow cytometry (gated on CD11c^+^ DC cells) (*n =* 3, mean ± SD). (I) Representative immunofluorescence images of tumours showing CD8^+^ T cells and DCs infiltration, scale bar = 200 *μ*m. (*n =* 3, mean ± SD). (J–L) Detection of IFN‐γ, TNF‐α, and IL‐6 in plasma after treatment (*n =* 3, mean ± SD). *p < 0.05, **p < 0.01, ***p < 0.001, by analysis of ANOVA with Turkey's post‐hoc test.

To verify the antitumor efficacy was mediated by improving the immune responses, we evaluated TME changes after ZnFe_2_O_4_‐PTX@CCM treatment, including cGAS/STING pathway‐related proteins, immune cells and cytokines. Results in Figure 7A and Figure S12 showed the expression of STING, p‐TBK1 and p‐IRF3 in tumour tissue were upregulated in ZnFe_2_O_4_@CCM group, and dramatically higher in ZnFe_2_O_4_‐PTX@CCM group. The phenomenon indicated that ZnFe_2_O_4_ could activate cGAS/STING pathway and PTX enhanced this effect, which was accordant with the previous results in vitro (Figure 5C). In addition, the mRNA expressions of downstream marker for cGAS/STING pathway (IFN‐β, TNF‐α) were significantly elevated in ZnFe_2_O_4_‐PTX@CCM group (Figure 7B,C).

Type I IFN (especially IFN‐β) is considered as the bridge between innate and adaptive immunity,^[^ ^32^, ^33^ ^]^ and can promote the maturation as well as migration of DCs. Flow cytometry analysis in Figure 7D,E demonstrated that ZnFe_2_O_4_‐PTX@CCM remarkably increase the proportion of mature DCs (CD11c^+^CD80^+^CD86^+^) (≈50%) compared with other control groups. Importantly, DCs isolated from tumour tissue treated by ZnFe_2_O_4_‐PTX@CCM possessed a higher capacity for antigen presentation (Figure S13). These results could be explained by the stimulation of cGAS/STING pathway originating from the synergism of Fe^3+^, Zn^2+^ and PTX. Professional antigen‐presenting DCs are essential for CD8^+^ T cell priming.^[^ ^34^ ^]^ We found that ZnFe_2_O_4_‐PTX@CCM induced a significant augment in the number of CD8^+^ T cells and CD4^+^ T cells (≈2.90‐fold and 1.67‐fold more than that of PBS group), contributing to the improved tumour killing activity (Figure 7F–H). Consistently, the immunofluorescence staining data presented a dramatically enhanced infiltration of CTLs (CD3^+^CD8^+^) and DCs in tumour after therapy (Figure 7I). Moreover, NKs (CD49^+^) that are crucial for innate immunity and promoted adaptive immune response^[^ ^32^ ^]^ were elevated 1.17‐3.95‐fold after various treatments, and ZnFe_2_O_4_‐PTX@CCM triggered nearly 18.70% NKs infiltration within TME (Figure S14).

Notably, a decrease of myeloid‐derived suppressor cells (MDSCs) and regulatory T cells (Tregs) was also observed in TME after ZnFe_2_O_4_‐PTX@CCM treatment (Figure S15). It might be because low‐dose PTX can not only kill Tregs through Bcl‐2/Bax‐mediated apoptosis, but also reduce MDSCs in tumours and promote their differentiation into DCs.^[^ ^35^, ^36^, ^37^ ^]^ Furthermore, inflammatory cytokines expression, such as IFN‐γ, interleukin‐6 (IL‐6), and TNF‐α were remarkably upregulated in ZnFe_2_O_4_‐PTX@CCM group (Figure 7J–L). Collectively, these results implied that ZnFe_2_O_4_‐PTX@CCM could enhance the antitumor immune response and improve the immunosuppressive TME.

### 2.7 ZnFe_2_O_4_‐PTX@CCM suppresses postoperative recurrence of B16F10 tumour

**FIGURE 8:**
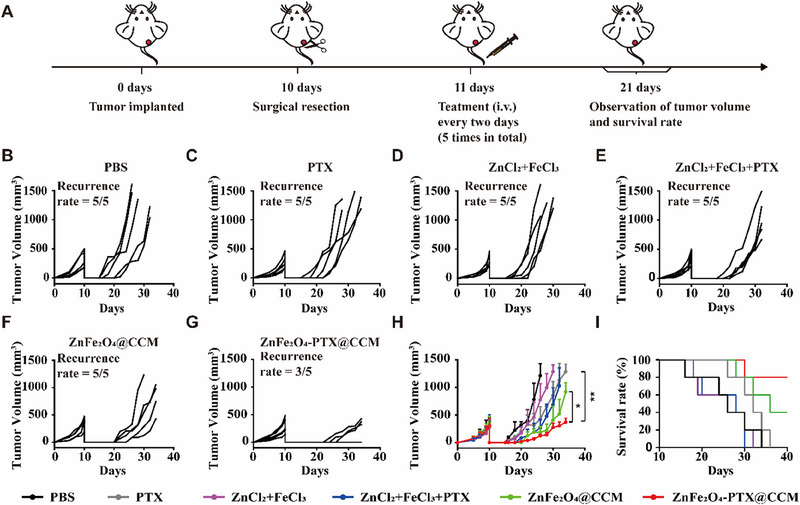
Postoperative recurrence inhibition of ZnFe_2_O_4_‐PTX@CCM. (A) Schematic illustrating of treatment on the tumour recurrence model. (B–G) Individual and (H) average tumour growth in different groups. Growth curve measurement was stopped when the first death occurred in the corresponding group (*n =* 5, mean ± SD). (I) The survival curves of mice after different treatments (*n =* 5). *p < 0.05, **p < 0.01, by analysis of ANOVA with Turkey's post‐hoc test.

Recurrence caused by residual tumour cells after surgery is always a challenge in clinic,^[^ ^38^ ^]^ but the adaptive immune response may provide long‐term protection against tumour recurrence.^[^ ^4^ ^]^ Therefore, we established a tumour recurrence model and assessed the local tumour growth after different treatments (Figure 8A). As can be seen in Figure 8B‐H and Figure S16, PTX, ZnCl_2_+FeCl_3_, ZnCl_2_+FeCl_3_+PTX and ZnFe_2_O_4_@CCM groups did not inhibit the recurrence. By contrast, tumour regeneration was controlled in ZnFe_2_O_4_‐PTX@CCM group, with undetected tumours in 2 of 5 mice and very slow tumour growth in the other 3 mice. In addition, compared with the free ions or drug treatment group, the median survival time for ZnFe_2_O_4_@CCM group was prolonged to 36 days, and the survival rate of mice in the ZnFe_2_O_4_‐PTX@CCM group was 80.00% in 40 days (Figure 8I). These results signified that ZnFe_2_O_4_‐PTX@CCM with homing targeting ability could optimize the synergistic effect of Zn^2+^, Fe^3+^ as well as PTX, and significantly enhance the immunotherapeutic efficacy.

## 3 CONCLUSION

In summary, the tailor‐designed ZnFe_2_O_4_‐PTX@CCM realized tumour cell targeting and ensured the induction of cytosolic DNA, improvement of cGAS‐DNA phase separation, and inhibition of mitotic process in tumour cells. Therefore, ZnFe_2_O_4_‐PTX@CCM programmatically initiated and enhanced cGAS/STING pathway in tumour cells, followed by promoting DCs maturation, increasing immune cells infiltration, and remodelling the immunosuppressive TME. The ZnFe_2_O_4_‐PTX@CCM achieved promising immunotherapeutic efficacy but did not emerge any side effects. This study provides feasible reference on the design of cGAS/STING enhancing formulations for tumour immunotherapy.

## CONFLICT OF INTEREST STATEMENT

The authors declare no conflicts of interest.

## Supporting information



Supporting information

Supporting information

## Data Availability

The data that supports the findings of this study are available in the supplementary material of this article.
