# Supplementary material for: Programmed initiation and enhancement of cGAS/STING pathway for tumour immunotherapy via tailor‐designed ZnFe2O4‐based nanosystem
Source: Exploration (Beijing). 2023 Nov 10;3(6):20230061. doi: 10.1002/EXP.20230061 (PMC10742191; doi:10.1002/EXP.20230061)
Supplement: Supplementary file 1 — Supporting information [file EXP2-3-20230061-s001.docx]

Supporting Information for

**Programmed initiation and enhancement of cGAS/STING pathway for tumor immunotherapy *via* tailor-designed ZnFe_2_O_4_-based nanosystem**

**Jing Yang^a, #^, Yuping He^a, #^, Meng Zhang^a^, Chenglin Liang^a^, Tongtong Li^a^, Tianjiao Ji^c,d^*, Mali Zu^c,d^, Xu Ma^c,d^, Zhenzhong Zhang ^a^, Chun Liang^b^, Qixu Zhang^e^, Youbai Chen^b,^*, Lin Hou****^a,^***

^a^ School of Pharmaceutical Sciences, Key Laboratory of Targeting Therapy and Diagnosis for Critical Diseases, Zhengzhou University, Zhengzhou 450001, China

^b^ Department of Plastic and Reconstructive Surgery, Chinese PLA General Hospital, Beijing 100853, China

^c^ CAS Key Laboratory for Biomedical Effects of Nanomaterials & Nanosafety, National Center for Nanoscience and Technology, Beijing 100190, China

^d^ University of Chinese Academy of Sciences, Beijing 100049, China

^e^ Department of plastic surgery. University of Texas MD Anderson Cancer Center, Texas 77030, USA

#These authors contributed equally to this work.

*****Corresponding Author:

Email addresses: jitj@nanoctr.cn (Tianjiao Ji), chenyoubai@301hospital.com.cn (Youbai Chen), houlin@zzu.edu.cn (Lin Hou)

**Supplementary Methods**

Materials

FeCl_3_·6H_2_O, ZnCl_2_, and ethylene glycol were purchased from Zhiyuan Chemical Reagent Co., Ltd (Tianjin, China). Crystalline sodium acetate was purchased from Yongda Chemical Reagent Manufacturing Co., Ltd (Tianjin, China). PTX was purchased from Dalian Meilun Biological Co., Ltd. Reactive Oxygen Species Assay Kit, BCA Protein Assay Kit, 4 ,6-diamidino-2-phenylindole (DAPI), Lyso-Tracker Red, ROS detection kit and Apoptosis Detection Kit were purchased from Beyotime Institute of Biotechnology (Jiangsu, China). Hoechst 33342 Stain solution and 3-(4,5-Dimethyl-2-thiazolyl)-2,5-diphenyl-2H-tetrazolium (MTT) bromide were purchased from Solarbio (China). Anti-Melanoma antibody gp100 (ab137078), Anti-dsDNA antibody (ab27156), goat anti-mouse IgG H&L Alexa fluor 488, (ab150117) were obtained from Abcam (Cambridge, UK). Prestained Protein Marker Ⅶ (8-195 kDa) (G2087) was purchased from Servicebio. TBK1/NAK (D1B4) Rabbit mAb (3504), Phospho-TBK1/NAK (Ser172) (D52C2) XP^®^ Rabbit mAb (5483), STING (D2P2F) Rabbit mAb (13647), IRF-3 (D83B9) Rabbit mAb (4302), Phospho-IRF-3 (Ser396) (4D4G) Rabbit mAb (4947), β-Actin (13E5) Rabbit mAb (4970) were obtained from Cell Signaling Technology. Anti-CD3-FITC (Biolegend, cat. no. 100204), anti-CD4-Alexa-Flour ^®^700 (Biolegend, cat. no. 100536), anti-CD8a-PerCP-Cy5.5 (Biolegend, cat. no. 100734), anti-Foxp3-Alexa-Flour ^®^647 (Biolegend, cat. no. 320014), anti-CD49b-PE-Cy7 (Biolegend, cat. no. 108922), anti-F4/80-PE (Biolegend, cat. no. 123110), anti-CD86-Brilliant Violet 605^TM^ (Biolegend, cat. no. 105037), anti-CD206-Brilliant Violet 421^TM^ (Biolegend, cat. no. 141717), anti-CD11c-Brilliant Violet 605^TM^ (Biolegend, cat. no. 117312), anti-CD80-PE (Biolegend, cat. no. 104707), anti-CD11b-Alexa-Flour ^®^488 (Biolegend, cat. no. 101217), anti-Gr-1- PE-Cy7 (Biolegend, cat. no. 108416), MHC-Ⅱ (Biolegend, cat. no. 107632) were purchased from Biolegend. RNA extraction kit was purchased from Bioteke. PrimeScriptTMRT reagent Kit with gDNA Eraser Kit and TB Green Premix Ex TaqTM II (Tli RNaseH Plus) were purchased from Takara.

Cell Lines and Animals

Murine melanoma cell line B16F10 was purchased from Wuhan Prosser Life Technology Co., Ltd. DC2.4 were obtained from Jinyuan Biological Co., Ltd (Shanghai, China). B16F10 and DC2.4 cells were cultured in Roswell Park Memorial Institute (RPMI) 1640 (Solarbio) medium with 10% FBS, penicillin (100 U/mL), streptomycin (100 U/mL), and 1% L-glutamine. The purchased normal cell lines (HUVEC) were grown in DMEM containing antibiotics (100 µg/ml penicillin and 100 UI/ml streptomycin) and FBS 10%. Incubation was performed at 5% CO_2_ and 37℃. C57BL/6 mice (6-8 weeks old, female, 18-20 g) were purchased from SPF Biotechnology Co., Ltd. (Beijing, China). All animal procedures were performed in accordance with the Guidelines for Care and Use of Laboratory Animals of Zhengzhou University. The animal laboratory's accreditation number is SCXK (YU) 2019-0004.

Preparation of ZnFe_2_O_4_

FeCl_3_·6H_2_O (3.0 mmol) and ZnCl_2_ (1.5 mmol) were dissolved in ethylene glycol (40 mL), followed by adding polyethylene glycol (2.0 g, molecular weight: 4000 kD) with stirring to form a clear solution. Then, crystalline sodium acetate (1.8 g) was slowly added to the mixture. The resulting mixture was vigorously stirred at 50 °C for 30 min to obtain a homogeneous brown solution. The solution was transferred to a stainless steel autoclave lined with Teflon, sealed and placed in a muffle furnace, and reacted at 160 °C for 16 h. Then, the solid product was collected with a magnet after the autoclave recover to the room temperature. After washing three times with ethanol, the samples were dried in a vacuum oven at 60 °C for 8 h.

Preparation of ZnFe_2_O_4_-PTX@CCM

ZnFe_2_O_4_ nanoparticles were prepared by a one-step solvothermal method.^[1]^ To obtain the PTX-loaded nanoparticles (ZnFe_2_O_4_-PTX), the prepared ZnFe_2_O_4_ (3.0 mg) was dispersed in deionized water (3 mL) at pH 7.4 to form a first mixed solution. PTX (6.0 mg) was dissolved in ethanol (1 mL) to form a second mixture. The second mixed solution was slowly added to the first mixed solution with the shaken at 37 °C for 24 h, and then centrifuged at 15000 g for 20 min. The supernatant was discarded, and the precipitate was washed with ethanol. The dry ZnFe_2_O_4_-PTX was obtained after placing it in the vacuum at 70 °C for 6 h.

CCM were extracted from B16F10 cells according to the reported procedure.^[2]^ The cell membrane precipitate obtained was quantified by using BCA protein quantitative kit, and stored at -80 ℃ for later use. To coat ZnFe_2_O_4_-PTX with CCM, a mixture of ZnFe_2_O_4_-PTX and B16F10 CCM was extruded through a 200 nm polycarbonate film at least 5 times.

Characterization of ZnFe_2_O_4_-PTX@CCM

The morphologies of ZnFe_2_O_4_ and ZnFe_2_O_4_-PTX@CCM were observed by TEM (JEM1200EX, JEOL, Japan). The hydrodynamic size and zeta potential of the nanoparticles were measured by Nano ZS Zetasizer (DLS, Zetasizer Nano ZS-90, Malvern, UK). The specific surface area and average pore diameter of ZnFe_2_O_4_ were detected by an automatic surface and porosity analyzer (BET, ASAP2460, Micromeritics, USA). The cell membrane protein composition of NPs was characterized by sodium dodecyl sulfate polyacrylamide gel electrophoresis (SDS-PAGE) and coomassie brilliant blue (Invitrogen, USA). The drug loading and encapsulation efficiency were determined by UV-vis spectrophotometer (UV-2550, SHIMADZU, Japan).

Degradation behavior of ZnFe_2_O_4_-PTX@CCM

ZnFe_2_O_4_-PTX and ZnFe_2_O_4_-PTX@CCM were dispersed in deionized water. PTX (0.5 mL, 1 mg) dispersion was transferred to a dialysis bag (MWCO=3.5 kDa), then immersed into PBS buffer solutions (30 mL, pH 7.4/6.8/5.0) containing 0.5% Tween 80 (W/V), and oscillated at 37 ℃ at 100 rpm. At the predetermined time points, the media solution (1 mL) was collected, and then all the media solution was changed to fresh one. The amount of released drug was measured by high performance liquid chromatography (HPLC). In addition, the contents of Zn and Fe ions released from supernatant were determined by inductively coupled plasma mass spectrometry (ICP-MS) (NWR-213, USA).

Cell viability assay

The viability of B16F10 cells and HUVEC cells were evaluated after treatment with different concentrations of ZnFe_2_O_4_-PTX@CCM for 24 h, using MTT method. In brief, an initial number of 1× 10^4^ cells were suspended in each well of 96-well plates. Cells were incubated with different concentrations of ZnFe_2_O_4_-PTX@CCM for 24 h. Supernatants were discarded and replaced with of MTT solution (5 mg/mL). Cells were kept for 3-4 h at 37 ˚C and Dimethylsufoxide (DMSO) solution was added. A microplate reader (Bio-rad, England) was used to read the optical density of each group and expressed as % of the control group.

*In vitro* cellular uptake and lysosome escape study

B16F10 cells were incubated with FITC-labeled ZnFe_2_O_4_@CCM in CLSM dishes. After co-incubation at different time intervals (1and 2 h), the nuclei were stained with Hoechst 33342, and lysosome was stained with Lyso-tracker red. The cellular uptake and lysosome escape was observed using CLSM (LEICA TCS SP8 STED, Germany).

CCM@ZnFe_2_O_4_-FITC was prepared using a similar method as that of PTX encapsulation. The prepared ZnFe_2_O_4_ (3.0 mg) was dispersed in deionized water (3mL, pH 7.4) to form the first mixed solution. Then, FITC (6.0 mg) was dissolved in ethanol (1mL) to form the second mixture. Subsequently, the second mixture was slowly added to the first mixture and stirred at 37 °C for 24 h. Finally, free FITC was removed by dialysis (MWCO = 8 kDa).

Intracellular accumulation of Zn and Fe test

B16F10 cells were seeded in petri dishes. After 24 h incubation, cells were treated with ZnCl_2_, FeCl_3_, and ZnFe_2_O_4_@CCM (Zn^2+^, 20 *μ*M; Fe^3+^, 40 *μ*M), respectively. After 6 h, the cell suspension was collected, the precipitation was collected by centrifugation, and PBS (1 mL) was added for washing. Then cell count was performed, and the number of cells in each group was recorded. The samples were centrifuged at 300 g for 5 min to collect the precipitate and transferred to 30% dilute nitric acid (2 mL). After probing in ice bath for 5 min (working for 3 s, with 5 s interval), the supernatant was centrifuged at 15000 g for 10 min, and the volume of solution in each group was quantified to 2 mL. The concentration of each sample was determined by ICP-MS (NWR-213, USA).

Intracellular ROS generation and detection

1×10^5^ cells /mL of B16F10 cells suspension (2 mL) were inoculated on 24-well plates. The media containing PBS, ZnCl_2_, FeCl_3_, PTX, ZnCl_2_+FeCl_3_, ZnCl_2_+FeCl_3_+PTX, ZnFe_2_O_4_@CCM and ZnFe_2_O_4_-PTX@CCM (Zn^2+^, 20 *μ*M; Fe^3+^, 40 *μ*M; PTX, 0.5 *μ*M) were added, respectively, and incubated for 6 h. According to the instruction of the ROS detection kit, DCFH-DA was added and incubated for 30 min. Fluorescence images were obtained at 488 nm.

Immunofluorescence of cytoplasmic dsDNA

Cells were seeded on confocal dishes at a density of 1×10^5^ cells/mL per well. Medium containing PBS, ZnCl_2_, FeCl_3_, PTX, ZnCl_2_+FeCl_3_, ZnCl_2_+FeCl_3_+PTX, ZnFe_2_O_4_@CCM and ZnFe_2_O_4_-PTX@CCM (Zn^2+^, 20 *μ*mol/L; Fe^3+^, 40 *μ*M; PTX, 0.5 *μ*M) were added, respectively, and incubated for 6 h. The cells were immobilized in 4% PFA in PBS at room temperature for 10 min. Cells were permeabilized with 0.5% TritonX-100 solution for 20 min, and then blocked with PBST containing 1% BSA and glycine (22.52 mg/mL) for 30 min. Cells were incubated with primary dsDNA antibody overnight at 4 °C, followed by the secondary antibody (goat anti-mouse IgG H&L Alexa fluor 488) for 1 h at room temperature.^[3]^ Nuclei were stained with Hoechst after washing. Images were acquired using a confocal microscope (LEICA TCS SP8 STED, Germany).

RT-qPCR analysis

Total RNA was extracted with an RNA extraction kit, and cDNA was transcribed into cDNA using a PrimeScriptTMRT reagent Kit with gDNA Eraser Kit. Quantitative RT-PCR was performed using TB Green Premix Ex TaqTM II (Tli RNaseH Plus) (*n* *=* 3). The primer sequences used for DNA amplification are listed in Table 2.

*In vivo* biodistribution evaluation

B16F10 tumor-bearing mice were treated by injection of NR-labeled nanoparticles at a dose of 4.0 mg/kg NR (*n* = 3). NR in ZnFe_2_O_4_-NR and ZnFe_2_O_4_-NR@CCM was loaded in a similar way as PTX encapsulation. Mice were sacrificed at the determined time after administration, and the accumulation of NR in tissues was detected by immunofluorescence staining.

To further evaluate the targeting property of ZnFe_2_O_4_-PTX@CCM in various cells, B16F10 tumor-bearing mice were sacrificed at 12 h after intravenous injection of free NR, ZnFe_2_O_4_-NR and ZnFe_2_O_4_-NR@CCM (NR, 4.0 mg/kg). The harvested tumors were cut into small pieces and lysed for 30 min at 37 ℃ in culture medium containing collagenase IV (1.0 mg/mL) and DNase I (0.2 mg/mL) to obtain single cell suspension. Samples were stained with antibodies (AF647 anti-CD11c, PE/Cy7 anti-CD80, PE anti-F4/80, PerCP/Cy5.5 anti-CD86 (BioLegend), anti-Melanoma gp100 antibody, and donkey Anti-rabbit IgG-AF594) for flow cytometry analysis.

Antitumor effect evaluation

Antitumor studies were performed using the B16F10 tumor mice. B16F10 cells (1 × 10^6^) suspended in PBS were injected subcutaneously into the right flank of each female C57BL/6 mouse. When the tumor volume reached ∼100 mm^3^, mice were randomly divided into 6 groups (*n* = 10) and injected with PBS, PTX, ZnCl_2_+FeCl_3_, ZnCl_2_+FeCl_3_+PTX, ZnFe_2_O_4_@CCM and ZnFe_2_O_4_-PTX@CCM at an equivalent dose of ZnFe_2_O_4_ (3.0 mg/kg) and PTX (3.5 mg/kg，PTX solution, which is a commercial formulation, was prepared by dissolving 200 mL of 12mg/mL PTX in ethanol with an equal volume of Cremophor EL, followed by sonication for 30 min) *via* the tail vein every two days (5 times in total). The tumor volume and body weight were recorded every day. The tumor volume was calculated as follows: V = (length × width^2^)/2. After mice were sacrificed, tumors were collected and weighed. The inhibition ratio (IR) was calculated as the following formula: IR (%) = [(Wc-Wt)/Wc] × 100%, where Wc and Wt represented for the average tumor weight of control groups and treatment groups, respectively. Survival time was monitored and survival curves were generated. In addition, all major organs were collected and examined by H&E staining.

In addition, postoperation recurrence inhibition effect was evaluated (*n* *=* 5). After tumor resection, mice were injected respectively with PBS, PTX, ZnCl_2_+FeCl_3_, ZnCl_2_+FeCl_3_+PTX, ZnFe_2_O_4_@CCM and ZnFe_2_O_4_-PTX@CCM at an equivalent dose of ZnFe_2_O_4_ (3.0 mg/kg) and PTX (3.5 mg/kg) *via* the tail vein every two days (5 times in total). The recurrent tumor size was measured every 2 days, and the experimental end point was defined as either death or a tumor size greater than 1500 mm^3^. Animals were euthanized when exhibited signs of imperfect health or when the size of their tumors exceeded 1500 mm^3^.

TUNEL staining

Tumor sections were fixed in 4% of paraformaldehyde for 20 min and washed with cold PBS. Then, the tumor slices were stained with TUNEL reaction mixture (50 *μ*L) for 60 min at 37 °C. Lastly, the cell nuclei were stained with DAPI and visualized by CLSM (LEICA TCS SP8 STED, Germany).

Flow cytometry analysis

Tumors collected from mice were divided into small pieces and homogenized in cold staining buffer to form a single-cell suspension in the presence of digestive enzymes (*n* *=* 3). Antibodies were used to stain cells following the manufacturer's instructions. Stained cells were detected on a flow cytometer (FACS Calibur, BD co., USA) and analyzed by FlowJo software (version 10.0.7, TreeStar).

Immunofluorescence

Tumor tissues were collected, cryosections were prepared, and different primary antibodies (CD3, CD8, CD11c, CD86, F4/80, CD86) were used to incubate overnight at 4 °C according to the manufacturer's instructions (*n* *=* 3). A fluorescence labeled secondary antibody (goat anti-mouse IgG (H+L)) was added. Images were acquired using CLSM, and quantitative analysis was performed using ImageJ.

Western blotting

Total protein from cells and tumor tissues was obtained by using radioimmunoprecipitation assay lysis buffer containing protease inhibitor cocktail. Equal amounts of protein from each group were separated by SDS-PAGE (*n* *=* 3). The gel is cut according to the molecular weight of the protein and then transferred to the polyvinylidene fluoride membrane, which can ensure the most accurate results of the protein on the same gel. After block with 5% nonfat milk for 1 h, the membrane was incubated with primary antibody overnight at 4 °C. Prestained Protein Marker Ⅶ (8-195 kDa) (G2087) was purchased from Servicebio. Antibodies used included β-Actin (CST, USA), TBK1/NAK (D1B4) (CST, USA), phospho-TBK1/NAK (Ser172) (CST, USA), IRF-3 (D83B9) (CST, USA), phospho-IRF3 (S396) (CST, USA), STING (CST, USA). Horseradish peroxidase-conjugated goat anti-rabbit (CST, USA) was used to detect bound primary antibody. Signals were detected by enhanced chemiluminescence reagents and analyzed by ImageJ software. In order to obtain clear and concise experimental results, the brightness of the image is adjusted by AJFramework software.

Cytokine detection

IFN-γ, TNF-α and IL-6 in plasma were detected by ELISA kit (MultiSciences) according to the instructions (*n* *=* 3). Plasma samples were isolated from mice after different treatments and diluted for analysis.

Safety evaluation

Healthy mice were treated with different formulations for evaluating the biosafety. Tissue sections of heart, liver, spleen, lung, kidney and tumor were stained with H&E to examine pathological changes. In addition, blood samples were collected from C57BL/6 mice treated with different preparations, and aminotransferase (ALT), alkaline phosphatase (ALP), creatinine (CREA) and blood urea nitrogen (UREA) levels were determined (*n* *=* 3). Body weights were recorded daily (*n* *=* 10).

Statistical analysis

Data were expressed as mean ± standard de*via*tion (SD) of at least three independent experiments. Comparison of parameters for more than three groups were performed by one-way analysis of variance (ANOVA) followed by Tukey’s significant different post-hoc test. Overall survival curves were generated using the Kaplan-Meier method and estimated by the long rank-test. All statistical analysis were conducted using or SPSS 26.0. P-values less than 0.05 were considered as significant difference.

References

[1] M.P. Reddy, A.M.A. Mohamed, *Microporous Mesoporous Mater.* **2015**, 215, 37-45.

[2] M. Fusciello, F. Fontana, S. Tahtinen, C. Capasso, S. Feola, B. Martins, J. Chiaro, K. Peltonen, L. Ylosmaki, E. Ylosmaki, F. Hamdan, O.K. Kari, J. Ndika, H. Alenius, A. Urtti, J.T. Hirvonen, H.A. Santos, V. Cerullo, *Nat. Immunol.* **2019**, 10, 5747.

[3] C. Vanpouille-Box, A. Alard, M.J. Aryankalayil, Y. Sarfraz, J.M. Diamond, R.J. Schneider, G. Inghirami, C.N. Coleman, S.C. Formenti, S. Demaria, *Nat. Immunol.* **2017**, 8, 15618.

**Supplementary Results**


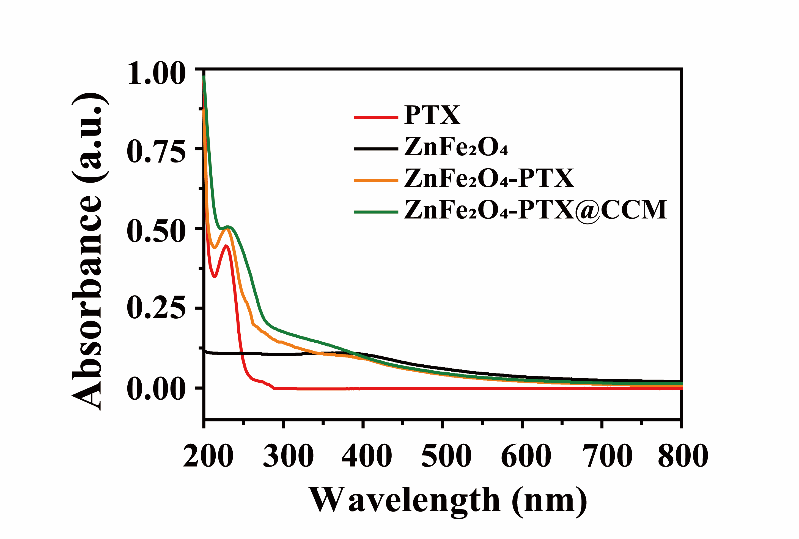


**Figure S1.** UV−vis absorption spectra of free PTX, ZnFe_2_O_4_, ZnFe_2_O_4_-PTX and ZnFe_2_O_4_-PTX@CCM.


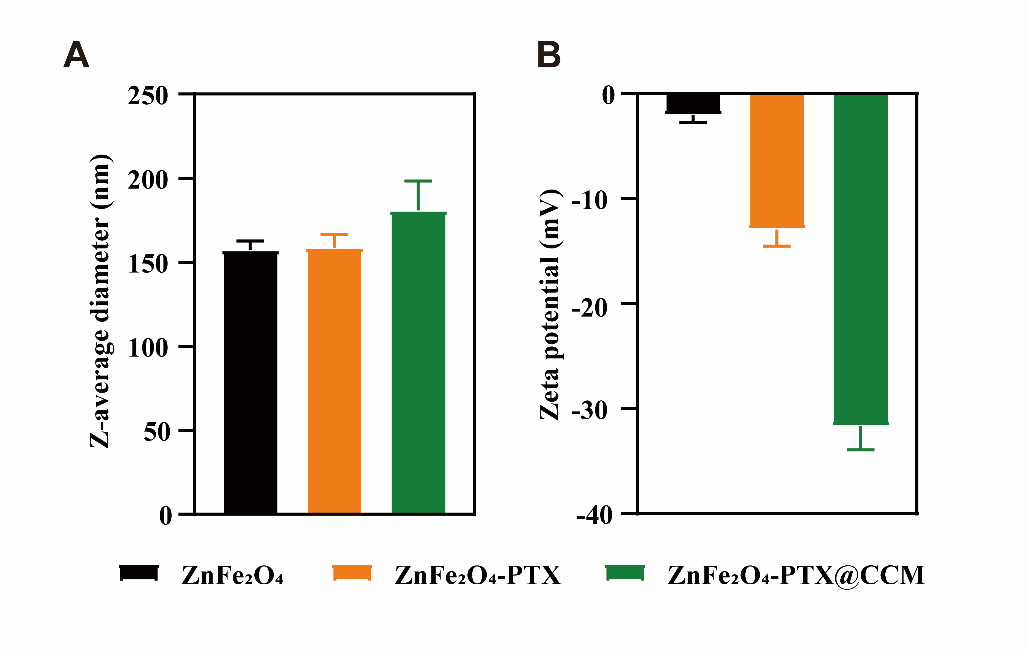


**Figure S2.** Size distribution (A) and zeta potential (B) of ZnFe_2_O_4_, ZnFe_2_O_4_-PTX and ZnFe_2_O_4_-PTX@CCM determined by DLS. Data are presented as mean ± SD (*n* *=* 3).


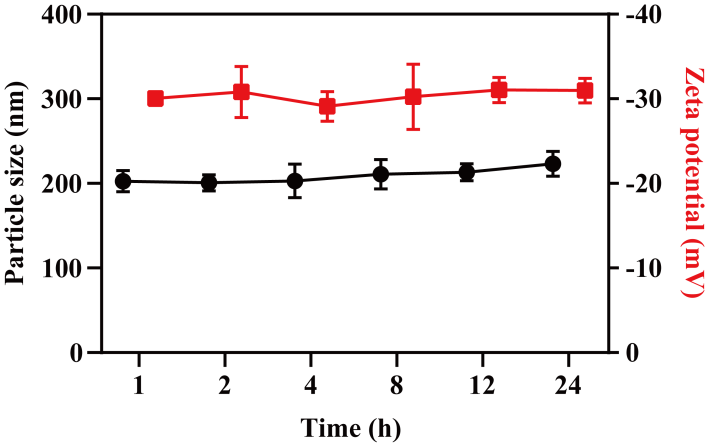


**Figure S3.** Stability of ZnFe_2_O_4_-PTX@CCM in PBS buffer determined by DLS. The black line shows the change in particle size and the red line shows the change in potential. Data are presented as mean ± SD (*n* *=* 3).


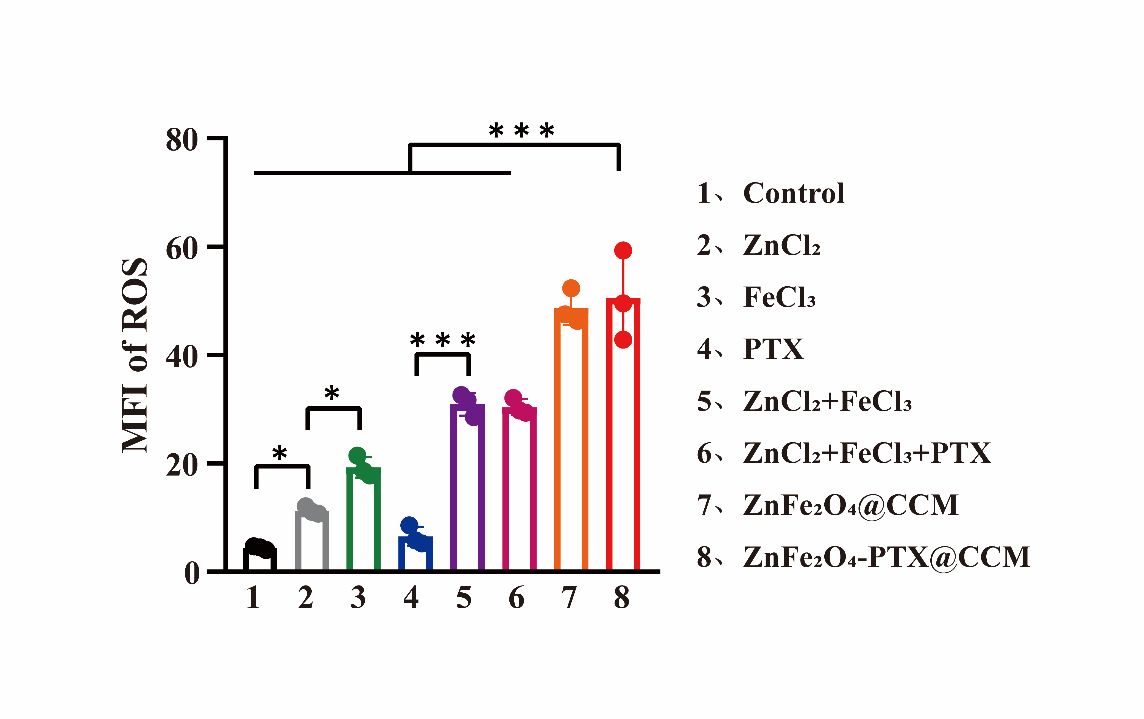


**Figure S4.** Quantitative calculation (Figure 5A) of fluorescent intensity (of ROS) in B16F10 cells from each formulation treated group. Data are presented as mean ± SD (*n* = 3).


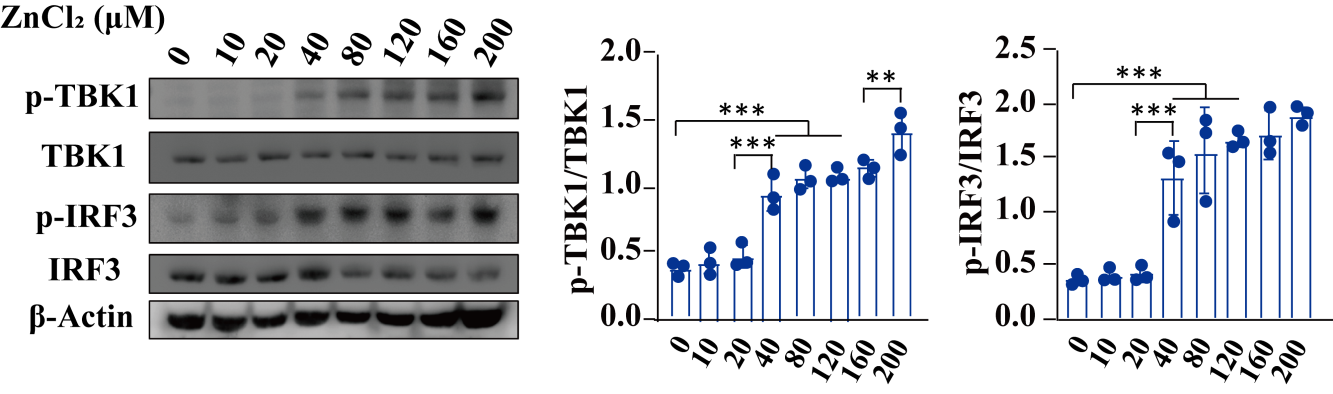


**Figure S5.** Western blot of B16F10 cells incubated with different concentrations of ZnCl_2_. Data are presented as mean ± SD (n = 3).


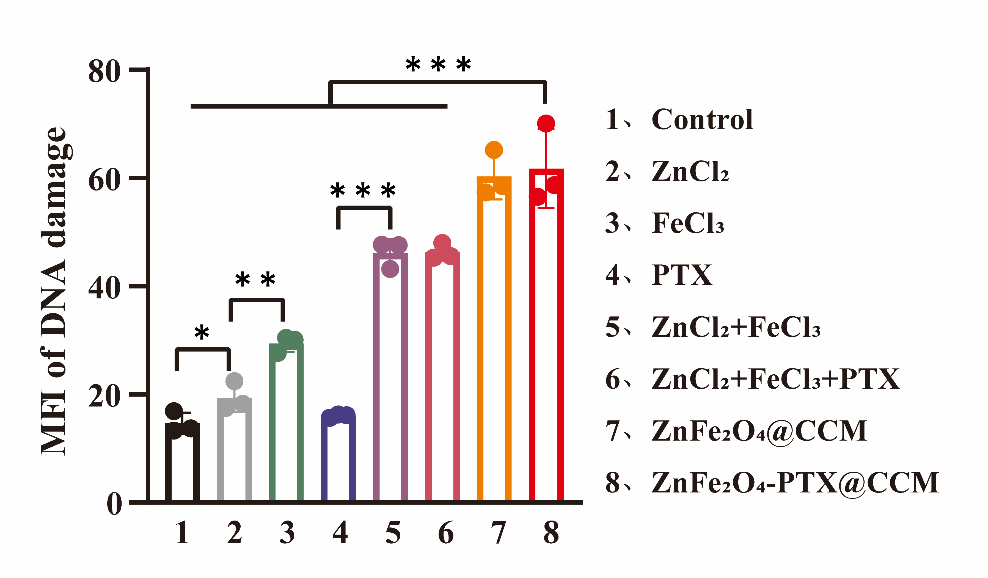


**Figure S6.** Quantitative calculation (Figure 5B) of fluorescent intensity from cytoplasmic dsDNA. Data are presented as mean ± SD (*n* = 3).


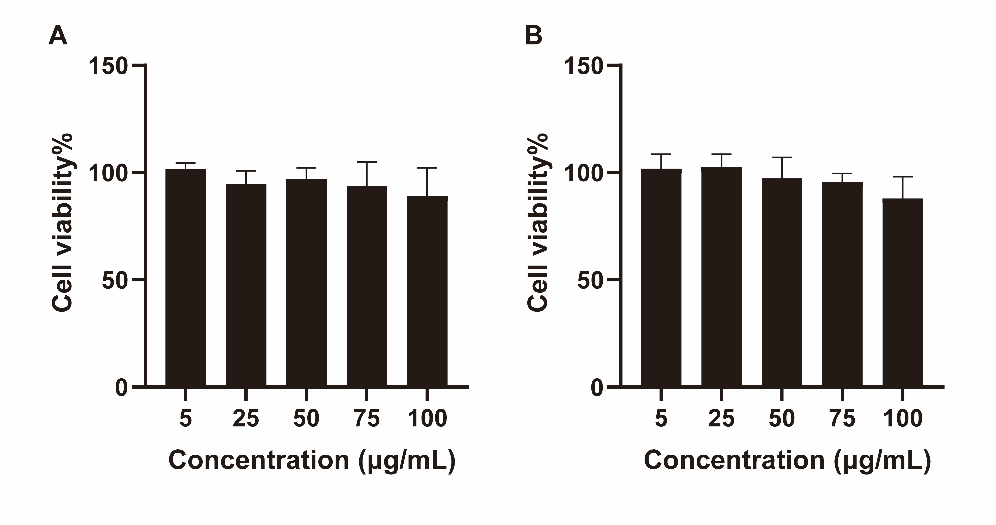


**Figure S7.** Cell viability of B16F10 cells (A) and HUVEC cells (B) after 24 h of incubation with ZnFe_2_O_4_-PTX@CCM at different concentration. Data are presented as mean ± SD (*n* = 5).


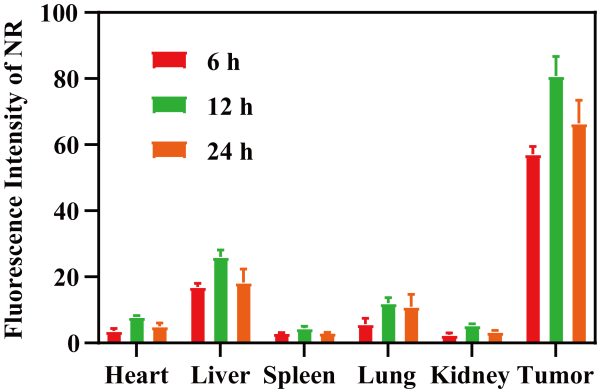


**Figure S8.** Biodistribution of ZnFe_2_O_4_-NR@CCM in B16F10 tumor-bearing mice. Data are presented as mean ± SD (*n =* 3).


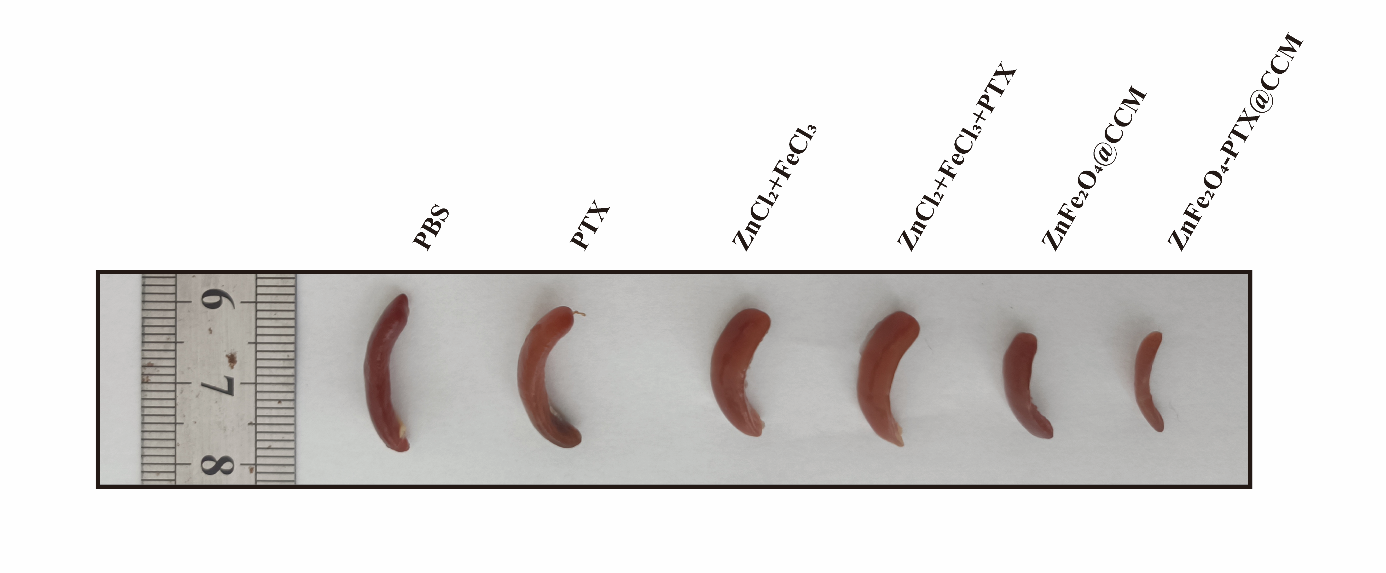


**Figure S9.** Spleen images and size evaluation from B16F10 tumor-bearing mice in each formulation treated group.


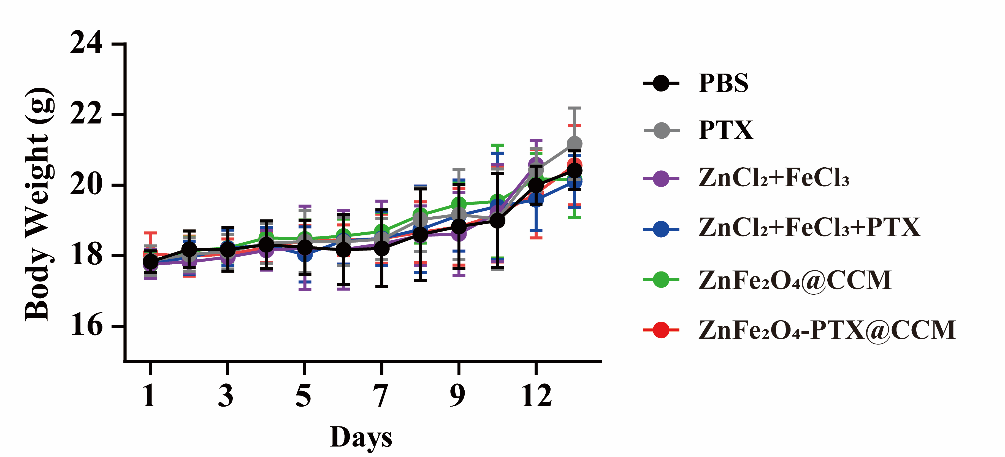


**Figure S10.** Body weight change of mice in each formulation treated group. Data are presented as mean ± SD (*n* *=* 10).


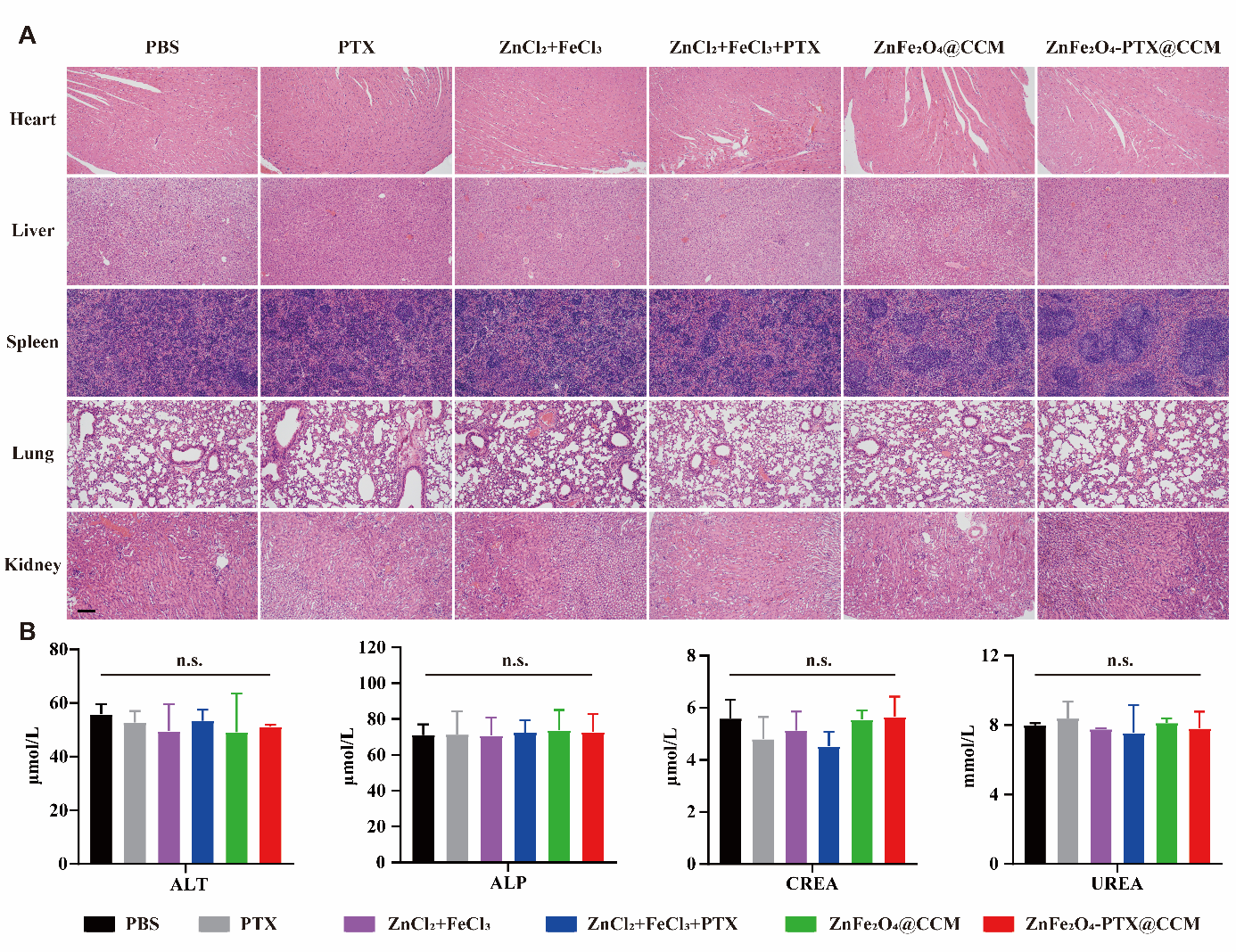


**Figure S11.** Biosafety evaluation of ZnFe_2_O_4_-PTX@CCM. A) H&E morphology evaluation of major organs, scale bar: 500 *μ*m. B) Blood biochemical parameters of healthy mice treated with different formulations. Data are shown as the mean values ± SD (*n* *=* 3). ns: no significant differences (*p* > 0.05).


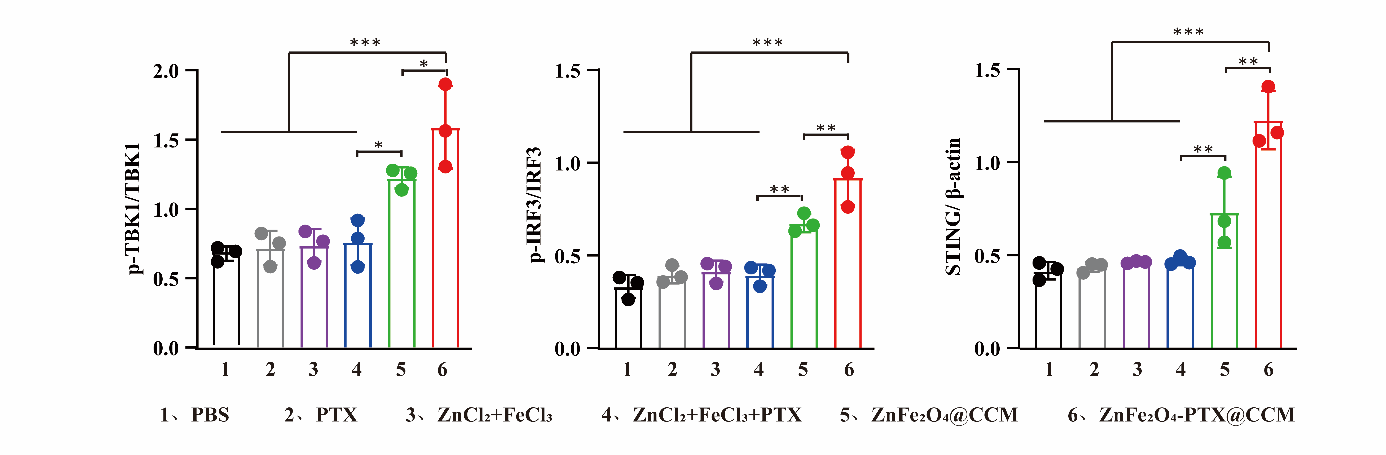


**Figure S12.** Semiquantitative analysis of WB bands in Figure 7A. Data are presented as mean ± SD (*n* *=* 3). **p* < 0.05, ***p* < 0.01, ****p* < 0.001, by analysis of ANOVA with Turkey’s post-hoc test.


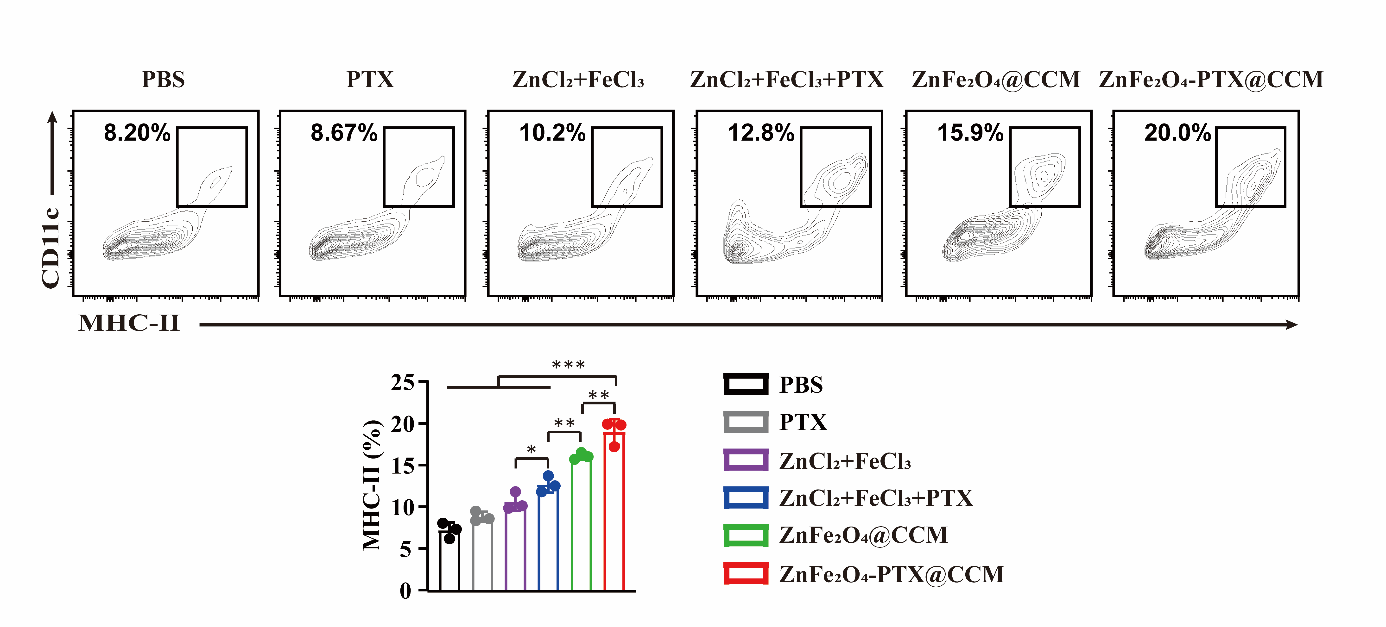


**Figure S13.** The expression of MHC-Ⅱ in tumor-infiltrating DCs was detected by flow cytometry. Data are presented as mean ± SD (*n* = 3). **p* < 0.05, ***p* < 0.01, ****p* < 0.001, by analysis of ANOVA with Turkey’s post-hoc test.


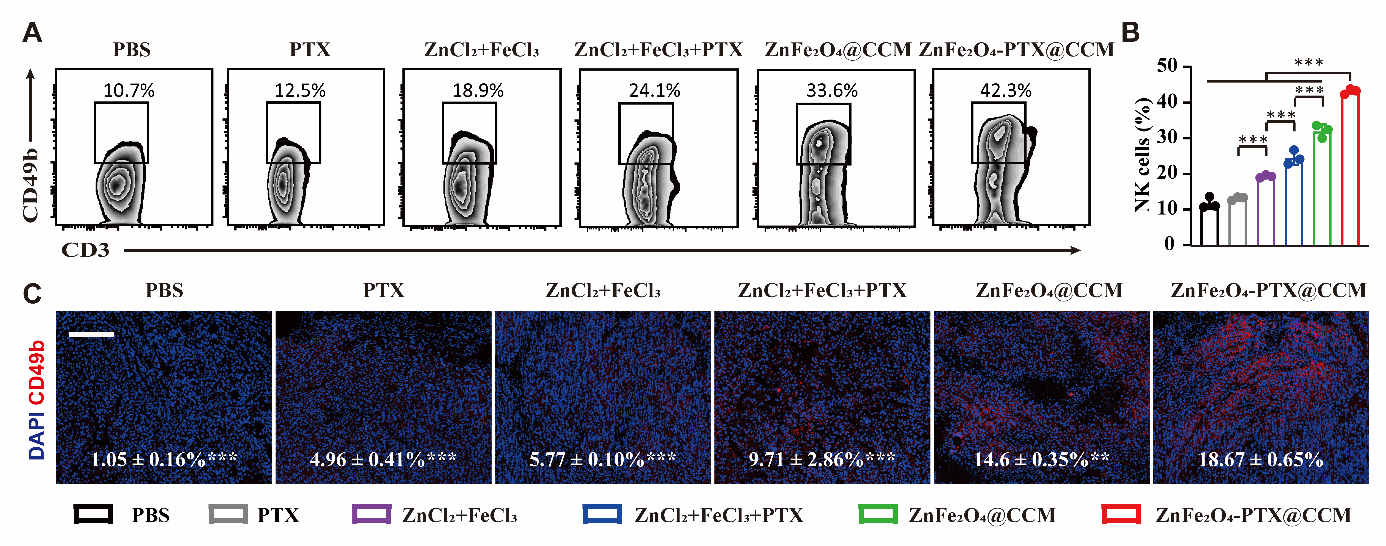


**Figure S14.** The infiltration of NKs in tumor. A, B) The infiltration of NKs (CD3^-^CD49b^+^) in tumor detected by flow cytometry. Data are presented as mean ± SD (*n* *=* 3). C) Representative immunofluorescence images of tumors showing infiltration of NK cells in tumor tissues in different treatment groups. Data are presented as mean ± SD (*n* = 3). **p* < 0.05, ***p* < 0.01, ****p* < 0.001, by analysis of ANOVA with Turkey’s post-hoc test.


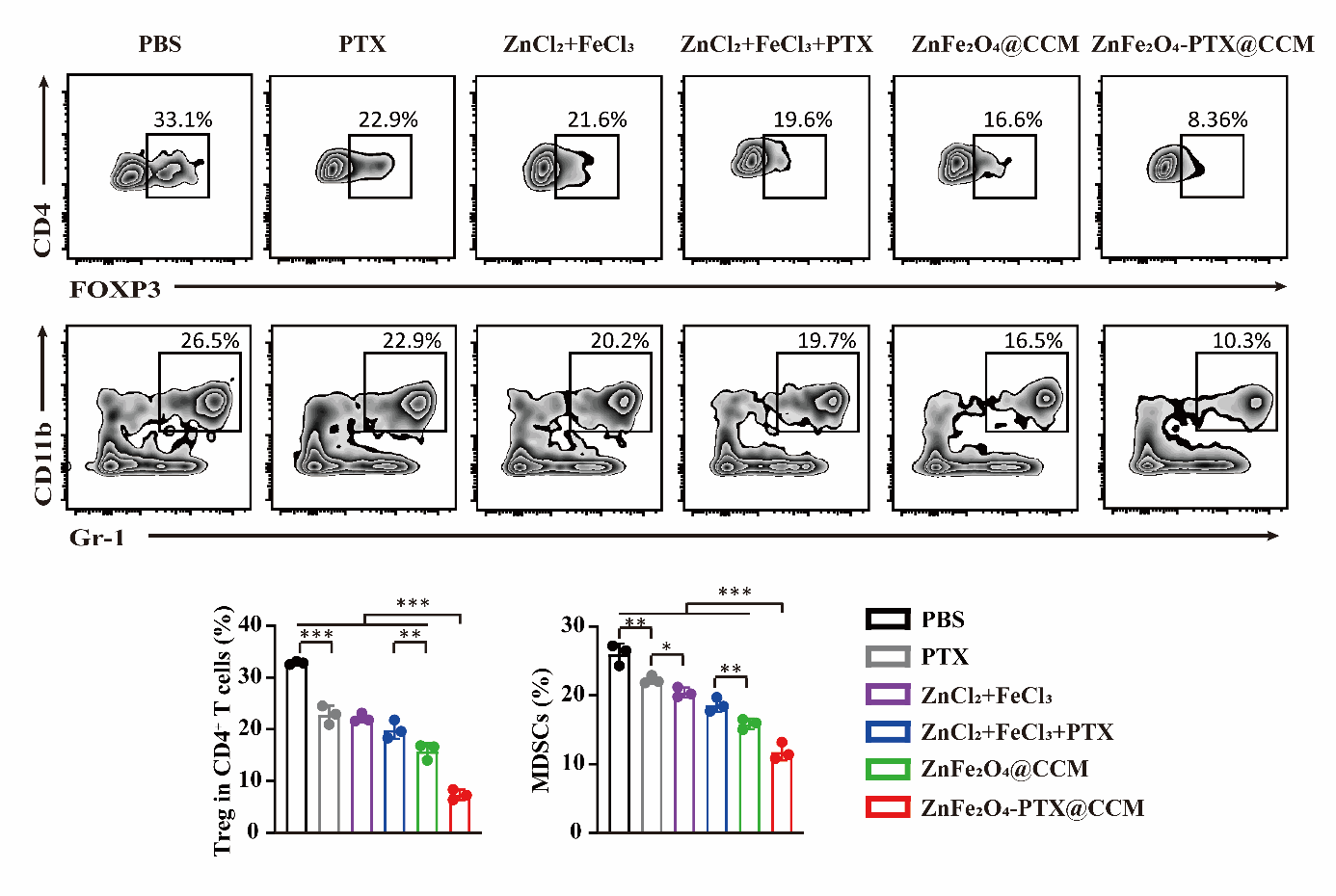


**Figure S15.** Percentage change of Tregs (CD4^+^Foxp3^+^) and MDSCs (CD11b^+^Gr-1^+^) detected by flow cytometry. Data are presented as mean ± SD (*n* = 3). **p* < 0.05, ***p* < 0.01, ****p* < 0.001, by analysis of ANOVA with Turkey’s post-hoc test.


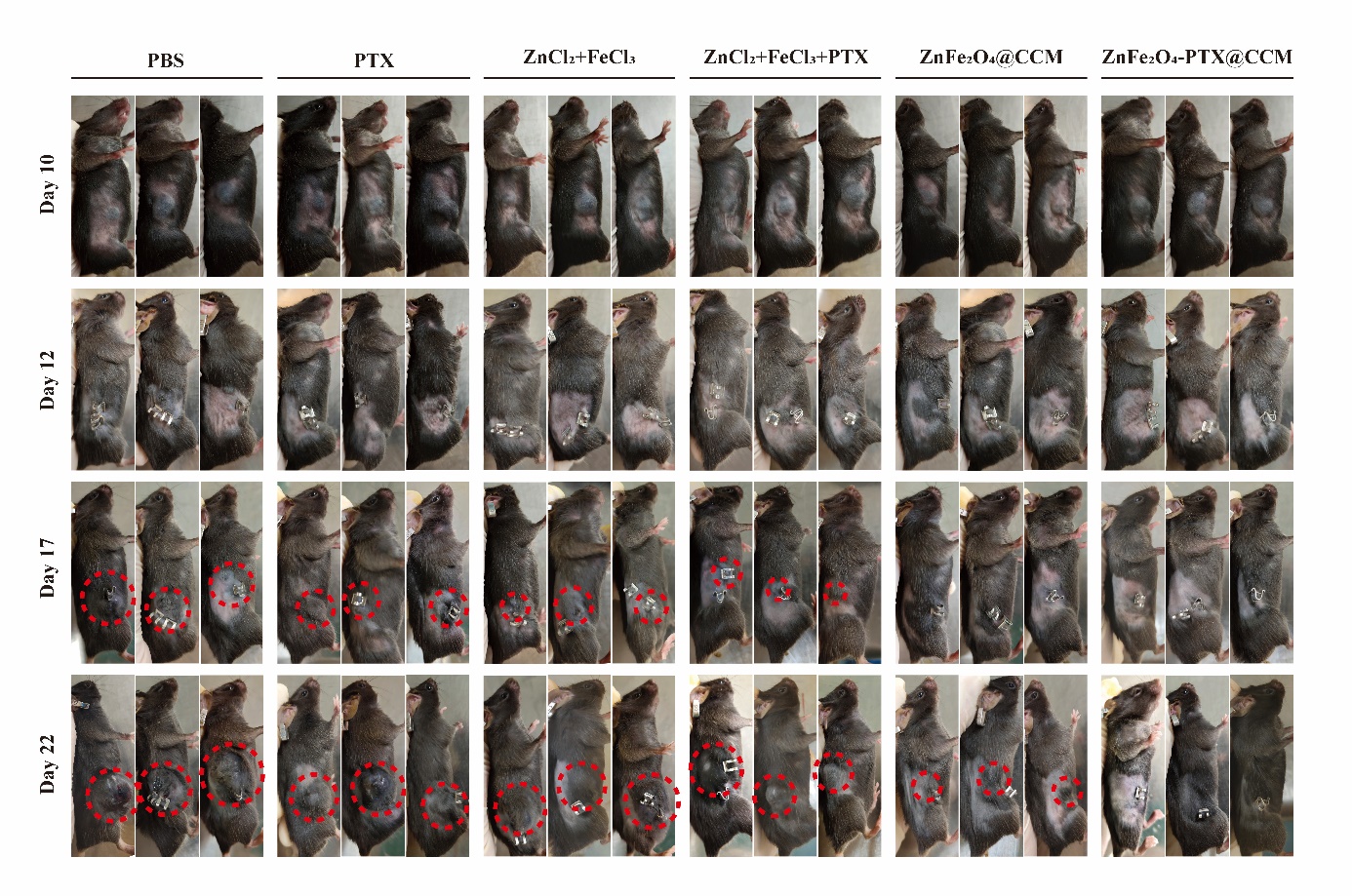


**Figure S16.** Representative pictures of B16F10 tumors bearing mice before and after primary tumor resection.

**Table S1.** Comparison of the BET specific surface area and BJH pore dimeter of the samples based on N_2_ adsorption-desorption isotherms.

| Sample | ZnFe_2_O_4_ | ZnFe_2_O_4_-PTX |
| --- | --- | --- |
| SBET (m^2^ g ^-1^) | 115.68 | 52.89 |
| Pore diameter (nm) | 10.69 | 8.02 |

**Table S2.** Primer sequences for PCR.

| Gene name | Sequence (5’-3’) |
| --- | --- |
| mIFN-β-F | CACAGCCCTCTCCATCAACT |
| mIFN-β-R | TCCCACGTCAATCTTTCCTC |
| GAPDH-F | TGATGGGTGTGAACCACGAG |
| GAPDH-R | TAGGGCCTCTCTTGCTCAGT |
